# A comparative bioinformatic analysis of *C9orf72*

**DOI:** 10.7717/peerj.4391

**Published:** 2018-02-19

**Authors:** Shalini Iyer, K. Ravi Acharya, Vasanta Subramanian

**Affiliations:** Department of Biology and Biochemistry, University of Bath, Bath, United Kingdom

**Keywords:** Amyotrophic Lateral Sclerosis (ALS), Genome, Frontotemporal Dementia (FTD), Bioinformatics, *C9orf72*

## Abstract

*C9orf72* is associated with frontotemporal dementia (FTD) and Amyotrophic Lateral Sclerosis (ALS), both of which are devastating neurodegenerative diseases. Findings suggest that an expanded hexanucleotide repeat in the non-coding region of the *C9orf72* gene is the most common cause of familial FTD and ALS. Despite considerable efforts being made towards discerning the possible disease-causing mechanism/s of this repeat expansion mutation, the biological function of *C9orf72* remains unclear. Here, we present the first comprehensive genomic study on *C9orf72* gene. Analysis of the genomic level organization of *C9orf72* across select species revealed architectural similarity of syntenic regions between human and mouse but a lack of conservation of the repeat-harboring intron 1 sequence. Information generated in this study provides a broad genomic perspective of *C9orf72* which would form a basis for subsequent experimental approaches and facilitate future mechanistic and functional studies on this gene.

## Introduction

Frontotemporal Dementia (FTD) and Amyotrophic Lateral Sclerosis (ALS) are two rapidly progressive debilitating neurodegenerative disorders. These two neurodegenerative diseases through their overlapping clinical, genetic, mechanistic as well as pathological features present a continuous disease spectrum albeit with two different manifestations (Lomen-Hoerth, Anderson & Miller, 2002; Andersen & Al-Chalabi, 2011). One of the unifying pathogenic signature appears in the form of a hexanucleotide repeat expansion (GGGGCC) in the non-coding region of the *C9orf72* gene (DeJesus-Hernandez et al., 2011; Renton et al., 2011). Wild type alleles carry between three and 30 of these hexanucleotide repeat units. People with the repeat expansion mutation, on the other hand, can harbor hundreds or even thousands of these repeat units (DeJesus-Hernandez et al., 2011; Renton et al., 2011). The hexanucleotide repeat expansion is unstable and repeat numbers have been shown to increase over generation (Vance et al., 2006).

Expanded hexanucleotide repeats in the non-coding region of *C9orf72* gene leads to a decrease in the levels of endogenous *C9orf72* protein (DeJesus-Hernandez et al., 2011; Donnelly et al., 2013; Belzil et al., 2013) alongside generating flawed nuclear RNA foci (Wojciechowska & Krzyzosiak, 2011; Van Blitterswijk, DeJesus-Hernandez & Rademakers, 2012), suggesting both gain of function as well as loss of function disease mechanism for *C9orf72* in ALS (Haeusler, Donnelly & Rothstein, 2016). Another major hypothesis that has been postulated to explain the disease mechanism is a protein gain-of-function mechanism. Studies have shown that depending on the reading frame, the hexanucleotide repeat unit can encode 5 types of repeat-associated non-ATG (RAN) translation products. These di-peptide repeat containing proteins, collectively called the RAN proteins, have been shown to contribute to the disease pathology by suppressing ribosomal RNA synthesis and impairment of stress granule formation (Ash et al., 2013; Gendron et al., 2013; Zu et al., 2013).

Highly sensitive bioinformatics analyses and structure-based homology methods predict *C9orf72* to be a part of a larger family of the differentially expressed in normal and neoplastic cells (DENN) domain containing proteins (Zhang et al., 2012; Levine et al., 2013) similar to Smith-Magenis chromosome region 8 (SMCR8), Folliculin (FLCN) and FLCN-interacting proteins 1 & 2 (FNIP-1/2). DENN-domain containing proteins are best known for the role they play in regulating intracellular membrane trafficking by functioning as guanine-nucleotide exchange factors (GEFs). Farg et al. (2014) showed physical interaction of *C9orf72* with Rab proteins 7 and 11 suggesting that *C9orf72* is likely to regulate vesicular membrane traffic by activating specific Rab-GTPase switches (Levine et al., 2013). *C9orf72* function has been implicated in inducing the formation of autophagosome (Webster et al., 2016) and in directing clearance of aggregated proteins through p62 (Sellier et al., 2016).

*C9orf72* was also shown to form a physically robust complex with SMCR8 (Amick, Roczniak-Ferguson & Ferguson, 2016) and WDR41 (Sullivan et al., 2016), which gets recruited to lysosomes, upon depletion of amino acids in the cell. This complex regulates signal transduction via mechanistic target of rapamycin complex 1 (mTORC1). Collectively, studies thus far suggest that *C9orf72* might function as a part of a larger complex and that members of the Rab-family might not be the only GTPases targeted by *C9orf72*. It is very likely that these complex functions of *C9orf72*, especially related to activating GTPase-switches as GEFs or even GAPs, might be under the tight control of nutrient availability.

Current studies have focused on the gain/loss of function mechanisms for *C9orf72* mediated ALS/ALS-FTD. Progress has also been made on characterizing the molecular/cellular functions of *C9orf72*, using biochemical approaches. In order to further our understanding of the *in vivo* function of *C9orf72* and the regulation of its expression using animal and cell culture models it is essential to have detailed information on its gene organization, synteny and its upstream regulatory elements. Here, we present the first detailed bioinformatics study that systematically extracts and analyzes the information on *C9orf72* gene using a comparative genome analysis approach. The goal of this article is to explore different aspects of this important molecule at a genetic level across different species and highlight the similarities/differences between them, especially between human and mouse. The mouse shares a near-identical genetic makeup with human, making it a very viable animal model to study not just how *C9orf72* contributes to ALS/FTD progression but also to understand the mechanistic and functional regulation of *C9orf72* in normal physiology.

## Materials and Methods

### Data collection

Genomic DNA, cDNA and protein sequences from a selection of species representing various taxonomic orders that have *C9orf72* were selected for comparison from the Ensembl database (http://www.ensembl.org). Table 1 lists the species compared along with their gene and transcript IDs. Data describing the various features of the genes are collated in Table 2 to provide easy assessment of similarities and differences between the species.

**Table 1:** Alternate splice variants of *C9orf72* gene in different species.

Species	Gene ID number	Transcript ID number
Human (*Homo sapiens*)	ENSG00000147894	ENST00000380003
		ENST00000379997
		ENST00000379995
		ENST00000461679
		ENST00000488117
Mouse (*Mus musculus*)	ENSMUSG00000028300	ENSTMUST00000084724
		ENSTMUST00000108126
		ENSTMUST00000108127
		ENSTMUST00000130538
		ENSTMUST00000142628
		ENSTMUST00000149138
		ENSTMUST00000156472
Fugu (*Takifugu rubripes*)	ENSTRUG00000001050	ENSTRUT00000002473
		ENSTRUT00000002474
		ENSTRUT00000002475
		ENSTRUT00000002476
Tetraodon (*Tetraodon nigroviridis*)	ENSTNIG00000005644	ENSTNIT00000008515
Zebrafish (*Danio rerio*)	ENSDARG00000011837	ENSDART00000015127
		ENSDART00000125180
		ENSDARKT00000126136
Stickleback (*Gasterosteus aculeatus*)	ENSGACG00000005564	ENSGACT00000007387

**Table 2:** Details of the intron-exon structure of *C9orf72* gene in different species.

		Human			Mouse			Fugu				Tetraodon	Zebrafish			Stickleback			
Chromosome location		9 Reverse strand			4 Reverse strand			Scaffold_396 forward strand				10 Reverse strand	13 forward strand			GROUP XV forward strand			
No of transcripts		5			7			4				1	3			1			
No of exons		11 exons 3,200 bp			11 exons 3,192 bp			10 exons 1,437 bp				11 exons 1,437 bp	10 exons 2,427 bp			10 exons 2,051 bp			
		5 exons 1,873 bp			10 exons 2,644 bp			9 exons 1,389 bp					9 exons 4,394 bp						
		5 exons 777 bp			10 exons 3,414 bp			7 exons 972 bp					11 exons 2,401 bp						
								9 exons 954 bp											
Length 5′ UTR (bp)		**1**	**2**	**3**	**1**	**2**	**3**	**1**	**2**	**3**	**4**		**1**	**2**	**3**				
		64	124	76	152	95	254	–	–	–	–	–	197	–	173	136			
Length of 3′ UTR (bp)		1,690	1,079	32	1,896	1,595	1,536	–	–	–	–	–	841	3,005	12,546	526			
Intron length (bp)	**1–2**	6,266	6,622	6,559	6,871	8,515	7,012	188	576	576	166	300	517	81	517	687			
	**2–3**	1,086	1,086	1,086	1,141	3,568	1,141	340	792	792	792	332	81	2,376	81	892			
	**3–4**	3,054	3,054	3,054	3,568	475	3,568	792	681	681	681	250	2,376	97	2,376	564			
	**4–5**	731	731	731	475	1,644	475	681	482	482	482	284	97	77	97	880			
	**5–6**	1,285			1,644	5,407	1,644	482	703	703	703	574	77	3,459	77	680			
	**6–7**	1,619			5,407	8,559	5,407	703	144	5	144	678	3,459	4,066	3,459	604			
	**7–8**	1,694			8,559	2,778	8,559	144	701		701	604	4,066	87	2	123			
	**8–9**	5,853			2,778	842	2,778	701	2,389		2,389	175	87	4,054	4,066	674	175	175	175
	**9–10**	1,983			842	116	842	2,389				658	4,054		87	2,130	658	658	658
	**10–11**	134			116							2,476			4,054		2,476	2,476	2,476
Transcription factor sites in intron 1		1,401	1,493	1,478	1,547	1,930	1,575	30	109	109	34	56	103	26	103	150			

### Phylogenetic analyses

Ensembl Compara provides cross-species resources and analyses, at both the gene and amino acid sequence level. Ensembl Compara was used to create the phylogenetic tree from a multiple sequence alignment of protein sequences that are representative of the longest protein-coding translation of the gene from all species compared. The program TreeDyn (Chevenet et al., 2006); (http://www.phylogeny.fr) was used to visualize and annotate the phylogenetic tree.

### Sequence alignments

The numbers and structure of alternative transcripts were acquired by searching the Ensembl database for each species of interest for *C9orf72*. Amino acid and cDNA sequences for *C9orf72* from all the species studied were aligned using ClustalW2 (Larkin et al., 2007), a multiple sequence alignment software using default alignment parameters (http://www.ebi.ac.uk/Tools/msa/clustalw2/). BOXSHADE program was used to shade regions of similarity in the aligned sequences (http://www.ch.embnet.org/software/BOX_form.html). Matcher (one of the EMBOSS programs) was used to locally align shorter sequences (Rice, Longden & Bleasby, 2000; McWilliam et al., 2013).

### Transcription factor binding site analysis

DiAlign TF and MatInspector (Cartharius et al., 2005) (http://www.genomatix.de/) were used to predict transcription factor binding sites within the nucleotide region encompassing the 5′-flanking region of the *C9orf72* gene (2,000 bp upstream) up to the end of intron 1 of human, mouse and *Fugu*. DiAlign TF builds alignments from gap-free pairs of similar segments of the sequences, looks for local similarities and then searches for common regulatory sequences in those aligned regions. TF binding site matches were identified (in both aligned and non-aligned upstream region of the three species) by MatInspector using Matrix Family Library Version 11.0.

MEME (Bailey & Elkan, 1994), Tomtom (Gupta et al., 2007) and GOMo (Buske et al., 2010), three motif-based sequence analysis tools from the MEME suite of programs, were used to analyze the upstream regulatory region in human, mouse and *Fugu*. MEME (Bailey & Elkan, 1994) was used to identify new ungapped motifs of recurring fixed-length patterns in the chosen nucleotide region. The motifs identified were searched against different databases of known motif (transcription factor families) using Tomtom (Gupta et al., 2007). GOMo was used to identify GO (gene ontology) terms associated with the DNA regulatory motifs identified by MEME (Bailey & Elkan, 1994).

### Comparative assessment of genomic synteny of *C9orf72*

To identify conserved syntenic regions, genes flanking C9orf72 in human, mouse and *Fugu* were annotated. Initial predictions were derived from the online server Cinteny (Sinha & Meller, 2007) (http://cinteny.cchmc.org/) and The Synteny Database (Catchen, Conery & Postlethwait, 2009). The programs identify syntenic regions across the genomes selected for comparison and analyse the extent of genome rearrangement using reversal distance as a measure. The predictions from these two programs were cross-referenced with those identified in the Ensembl database.

## Results

Comparative genomics, by virtue of utilizing evolutionary conservation across related species, provides a powerful approach for identifying functional elements within a genome. Genomic features including gene sequence, gene order, regulatory elements and other structural genomic landmarks of the *C9orf72* gene from different organisms were analyzed to study basic genetic and phylogenetic profiles and identify the elements that are conserved between species along with those that are unique.

Comparative analysis was carried out mainly on human, mouse and *Fugu* (Table 1), although in some of the analyses other vertebrate species were also compared in order to gain a more thorough insight. The human, mouse and *Fugu* were chosen because the human is the only species where the disease-causing repeat has been reported, the mouse gene since it has close genetic and physiological similarity to humans and the compact *Fugu* genome contains the same basic vertebrate blueprint as the human genome in an entity seven times smaller, making it an important resource for comparative genomics and to identify conserved regulatory elements.

### Gene location

**Figure 1:**
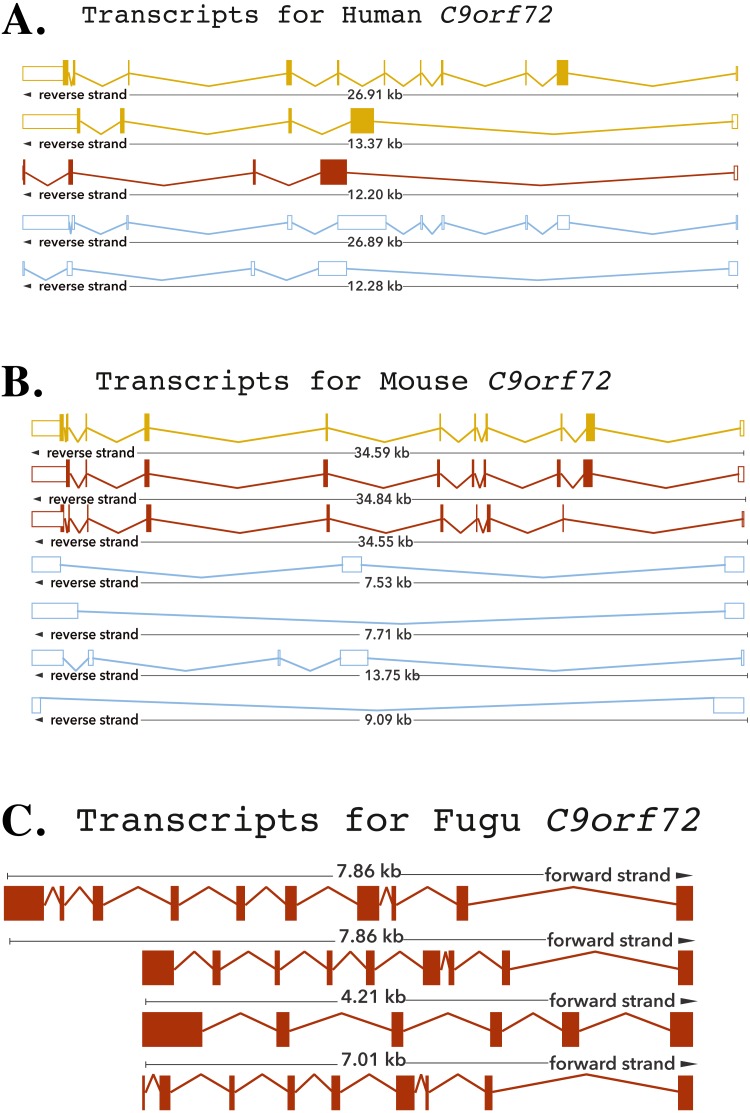
Cartoon representation of the *C9orf72* gene structure. Cartoon representation of the alternative splicing transcripts of *C9orf72* in human (A), mouse (B) and *Fugu* (C). Exons are shown as boxes and lines connecting the boxes represent introns. Filled boxes are coding sequence, and empty, unfilled boxes are UTR (UnTranslated Region). The coloring scheme follows that used by Ensembl. A red transcript comes from either the Ensembl automatic annotation pipeline or manual curation by the VEGA/Havana project. A gold transcript is identical between the Ensembl automatic annotation pipeline and manual curation by the VEGA/Havana project. Non-coding transcripts are coloured blue.

The human *C9orf72* gene is located on the short (p) arm of **c**hromosome **9** (cytogenic position 21.2) **o**pen **r**eading **f**rame **72**, from base pairs 27,546,542 to 27,573,863 (DeJesus-Hernandez et al., 2011; Renton et al., 2011). The gene, encoded on the minus strand of chromosome 9, consists of 11 exons that through alternative splicing are transcribed into 5 mRNA splice variants (DeJesus-Hernandez et al., 2011; Renton et al., 2011) (Fig. 1). The mouse gene, located on chromosome 4 on the reverse strand, like its human counterpart, also consists of 11 exons (Fig. 1). The *Fugu C9orf72* gene differs from the human and murine gene in that it is encoded on the forward strand of the chromosome (scaffold_396) and comprises of 10 exons (Fig. 1). Although, *Tetraodon* has an even smaller genome than the *Fugu*, it was not considered here because the annotation of the *Fugu* gene entry in Ensembl is believed to be more complete.

### Conservation of *C9orf72* in vertebrates

**Figure 2:**
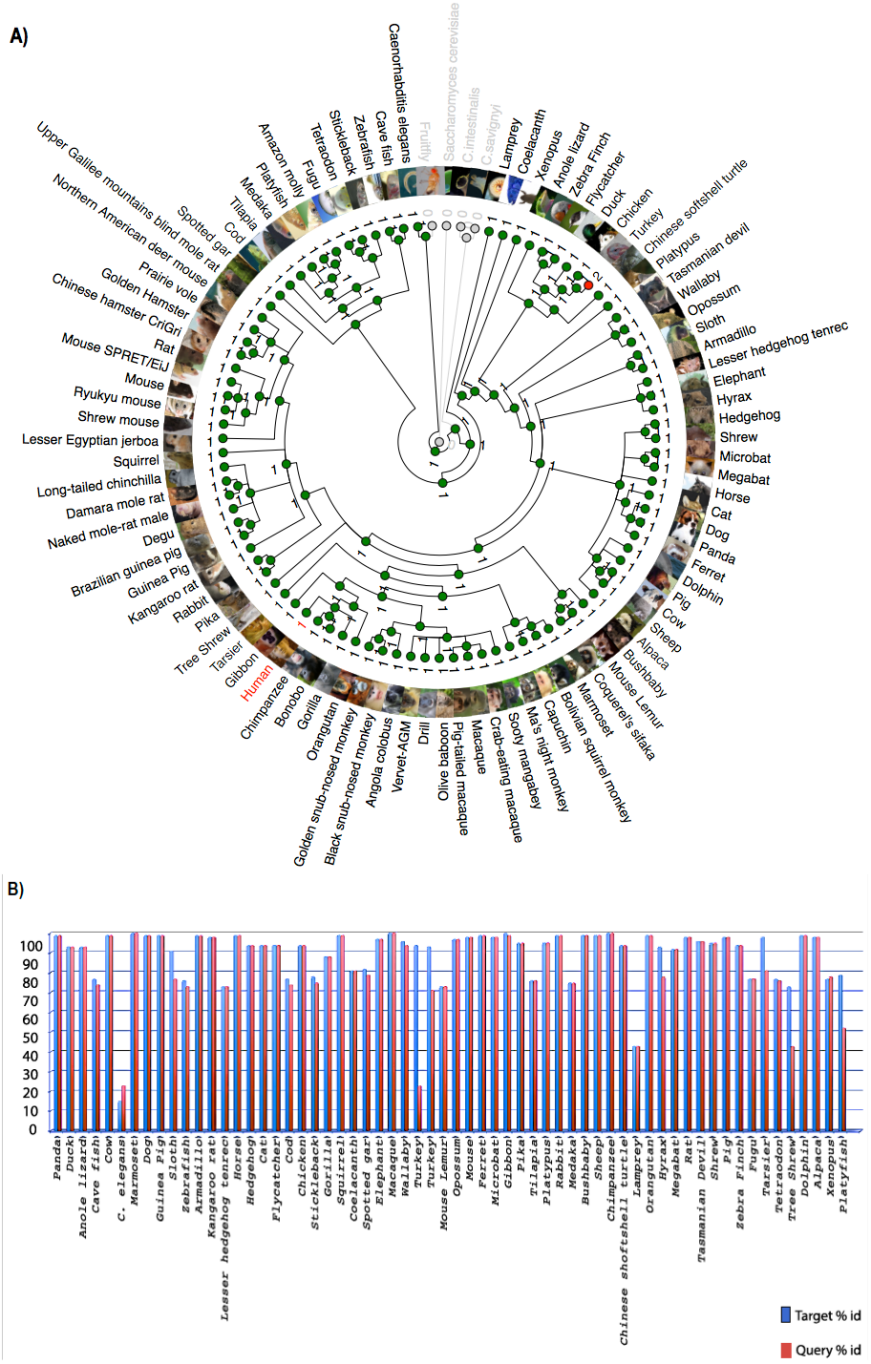
Phylogenetic analysis of *C9orf72*. (A) A rooted phylogenetic tree of all the species that express *C9orf72* generated by the Ensemble Compara server. The tree was drawn using TreeDyn (Chevenet et al., 2006). The branch-length scale represents substitutions per base pair. (B) A graph representing the percent of identical amino acids in the orthologues inferred from the gene trees constructed by Ensembl. The query % ID refers to the identity at the amino acid level of the gene of interest when compared with the orthologue in question. Target % ID refers to the percent of identical amino acids in the orthologue compared with the gene of interest. Both images have been downloaded from the Ensembl server.

Zhang et al. (2012) reported that *C9orf72*, a DENN-domain containing protein, could be traced all the way back to the last eukaryotic common ancestor. They also identified *C9orf72* homologues in protozoans and other species outside of the metazoa. Since the focus of this report is on understanding the degree of conservation of *C9orf72* amongst eukaryotes with particular emphasis on vertebrates, we looked at the phylogeny of *C9orf72* from this perspective. The phylogenetic tree we constructed for the analysis of the evolution of *C9orf72* gene amongst eukaryotes (Fig. 2A) revealed that this gene is absent in four species: *Ciona intestinalis, Ciona savignyi, Drosophila melanogaster and Saccharomyces cerevisiae*. Interestingly, a similar analysis conducted by us (Figs. S1–S3) for some other ALS risk factors such as angiogenin (*ANG*), Interferon Kappa (*IFNκ*) and NADH:Ubiquinone Oxidoreductase Subunit B6; (*NDUFB6*) reveals that these same genes are also absent in the four species mentioned above. At present it is unclear as to the significance of these findings.

It is worthwhile to note that *C9orf72* does not have any paralogs in vertebrates and there is no evidence of gene family expansion or contraction events that have affected this gene. The *ρ*-value for the *C9orf72* gene family was calculated to be 0.9410, suggesting that this gene has not gone through any significant gene gain or gene loss event during the course of vertebrate evolution. *C9orf72* appears to be an ancient gene and the presence of an ortholog in *Caenorhabditis elegans* suggests that it must have been present in the last common ancestor of all vertebrates. It also appears that *C9orf72* originated early in eukaryotic evolution since most extant eukaryotes possess only a single copy of the gene.

Statistically significant similarity between two protein sequences is frequently used to infer homology and therefore a common functional role. From the PSI-Blast (Altschul et al., 1997) results, all primate C9orf72 protein sequences have a pairwise identity of 99% with the human sequence and the mammals listed have alignment scores between 98% and 96% (Fig. 2B). The marsupials listed have scores of 94–96%, the birds have 93% and reptile proteins score 92%. *Xenopus laevis* scores 85% and the fish have scores ranging between 72–77%. This relatively high degree of protein similarity between species that are evolutionarily distant suggests there is a high degree of functional constraint on the protein. The residues are conserved all through the length of the protein suggesting that *C9orf2* functions as a single domain protein. *C9orf72* isoform containing 481 amino acids seems to be the major isoform expressed across all species. Given the high degree of sequence homology at the protein level and the consequent functional restraint on the protein, we wanted to know if this homology is reflected at the genetic level as well. We started by looking for conservation at the chromosome level followed by comparison of the different elements of the gene.

### Synteny

Chromosome level comparison of the genes/markers between human and mouse revealed that almost 50% of the genes/markers were common to both species. Cinteny (Sinha & Meller, 2007) identified 19 syntenic blocks and computed a reversal distance of 5 (the minimum number of reversals to translate from one genome to another). Analysis of synteny around the *C9orf72* loci showed that the gene is present in a conserved region of size 1.4 Mb in human (chromosome 9), whereas its mouse orthologue is present in a conserved region of size 2.1 Mb (chromosome 4).

**Figure 3:**
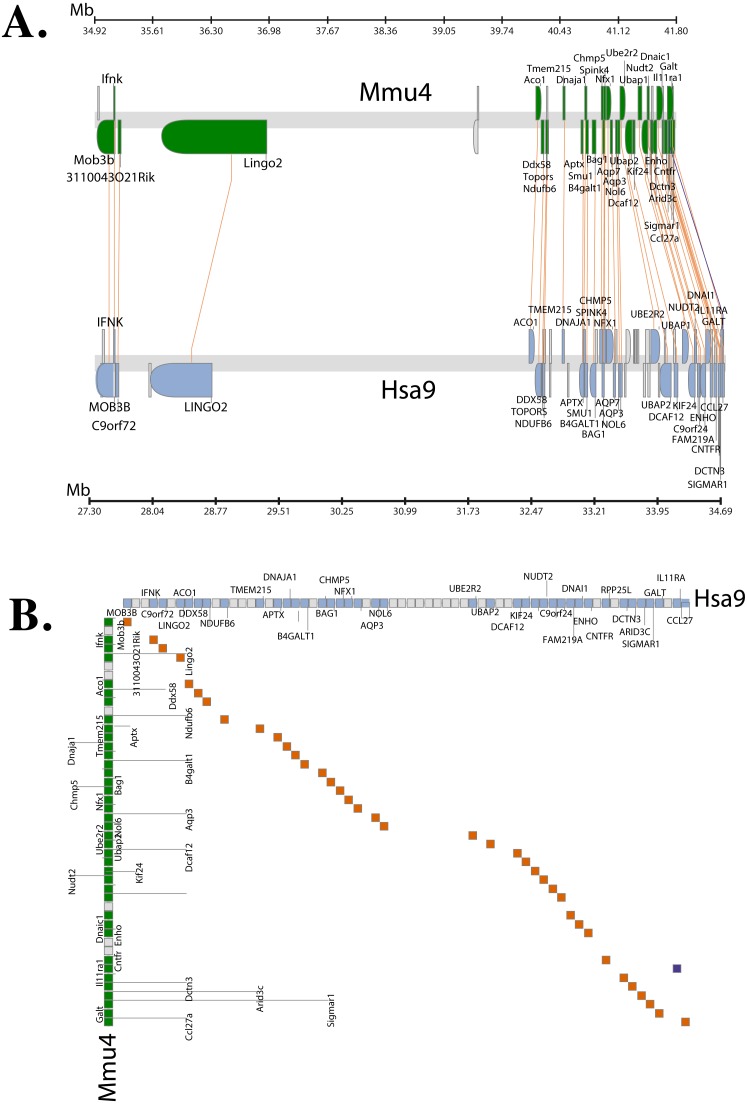
Conserved Synteny between human and mouse genomes. Synteny Database was used to detect the region of conserved synteny (Catchen, Conery & Postlethwait, 2009). (A) A trace image of the region of conserved synteny between human and mouse genomes. The image, created using a 25-gene sliding window, displays 39 orthologous pairwise clusters. The genes are displayed on their physical locations on the chromosomes, showing them on the proper strand, actual length, and in the proper orientation. Lines connect orthologs together. Genes colored grey are peripheral genes that are located within the same region of the cluster, but are not members of the cluster. (B) A gene homology matrix representing homology of the gene clusters between the chromosomal segments in human and mouse. Genes from the human cluster are placed along the *X*-axis of the plot while the genes from the murine cluster are placed along the *Y*-axis. Orthologs are marked at their intersection with well-conserved synteny shown as yellow boxes in a diagonal line in the plot and inversions of genes between the two chromosomes shown perpendicular to one another (blue boxes).

The Synteny Database (Catchen, Conery & Postlethwait, 2009) was also used to identify syntenic regions between human and mouse (Fig. 3A). The synteny trace built by the software covered a region of 7.4 Mb in human (chromosome 9) and 7.9 Mb in mouse (chromosome 4). Concomitant with the larger syntenic region, the software detected an orthologous pairwise cluster comprising of 39 gene pairs (Table S1) that are conserved between the source (human) and the outgroup (mouse) genomes. The gene homology matrix (Fig. 3B) showed collinear regions (diagonal lines yellow squares) representing homologous genes between the human and mouse chromosomes. The gaps in diagonal regions (shown as blue squares) correspond to insertions that have arisen either through translocation or deletions of genes. Neither the two programs used above nor Ensembl has an annotated genome for *Fugu*. In order to compensate for the lack of an annotated *Fugu* genome, we used Ensembl to calculate syntenic regions from pairwise whole genome alignments of the human genome with stickleback, *Tetraodon* and zebrafish genomes. The syntenic regions calculated using Ensembl are in agreement with those detected by Cinteny and The Synteny Database.

### Alternative splicing

The human *C9orf72,* through alternative splicing is transcribes five mRNA splice variants (Fig. 1). Of these, three transcripts are protein-coding variants with a validated polyadenylation tail at the 3′ end. The presence of the long and short isoforms of *C9orf72* has been validated using isoform-specific antibodies (Xiao et al., 2015; Davidson et al., 2017). Improved detection using these antibodies helped reveal distinct subcellular localisation of the two protein isoforms as well as showed that they had distinct biochemical profiles (Xiao et al., 2015; Davidson et al., 2017). The remaining two spliced variants are processed transcripts but without an annotated ORF. It is currently assumed that these transcripts do not translate into viable protein products (DeJesus-Hernandez et al., 2011; Renton et al., 2011).

The existence of different C9orf72 splice variants of course will need to be experimentally validated by testing these antibodies on *C9orf72* knock-in or knock-out tissues. In mouse, there are seven mRNA splice variants of which three are predicted to be protein coding and four not translated. Amongst these four variants of the mouse, two are predicted to undergo nonsense-mediated decay, as they are believed to retain intronic sequence relative to other coding variants. Alternative splicing of the *Fugu* gene is predicted to produce four transcription variants all of which are presumed to be translated to protein products (Fig. 1).

The longest mouse and human transcripts have a pairwise identity of 78%, the human vs. *Fugu* scored 70% and mouse vs. *Fugu* scored 74%. In human transcript 1, the start site is before exon 1 whereas in transcripts 2 and 3 it is found following exon 1—resulting in alternatively spliced transcripts that vary in their sequences (Fig. 1). The hexanucleotide repeat, GGGGCC, the expansion of which has been shown to be the pathologic link between ALS and FTD, is located in the core promoter region of human *C9orf72* splice variant 1 and in intron 1 of transcript variants 2 and 3. In mouse, all of the variation is found prior to exon 3 apart from one, short, additional sequence in the final exon of transcript 3. In *Fugu*, there is little sequence and structural variation. Transcripts 1 and 2 are almost identical to each other except for the addition of a short sequence between exons 1 and 2 in transcript 1. Exon 1 in transcripts 2 and 4 are quite different in *Fugu*. Other than that, there are no differences in these two transcripts. Transcript 3 is the most unique in *Fugu* (Fig. 1).

### Conservation of intron 1

The disease causing hexanucleotide repeat expansion occurs in intron one of *C9orf72* in humans. Since it has been suggested that the repeat expansion may affect gene expression of *C9orf72* (DeJesus-Hernandez et al., 2011; Renton et al., 2011) it is necessary to establish whether there is any conservation of intron 1 sequences as this would have implications when creating animal models.

**Figure 4:**
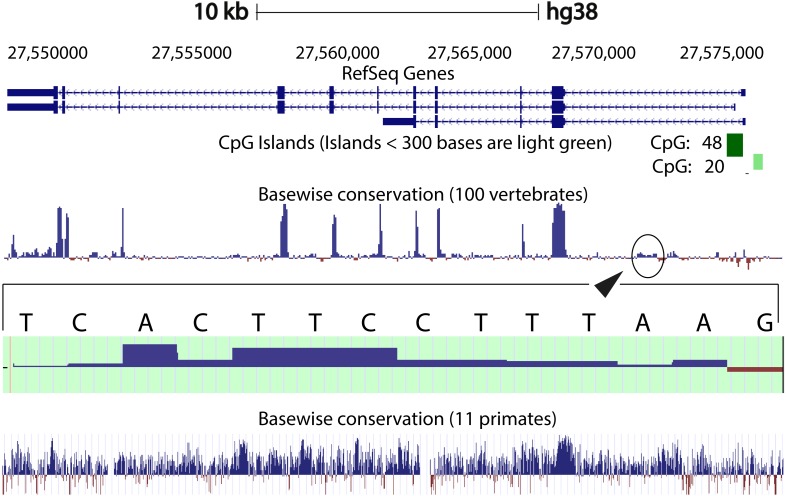
Genome graph for human *C9orf72*. Schematic representation of the genome graph of *C9orf72* as displayed by the UCSC Genome Browser (Kent et al., 2002). The figure shows basewise conservation of 100 vertebrates as well as 11 primates. Blue graphs represent conserved sites whilst red graphs represent faster evolving regions. The predicted CpG islands are also shown in green. The inset shows a region of ˜130 bp of higher conservation in Intron 1 with a transcription factor match to ETS1.

We compared the intron 1 sequences from different species. High levels of sequence conservation in noncoding DNA sequences compared between species can be interpreted as evidence for functional constraints. We found that there are no clear conserved sequences generated by aligning the intron sequences of all the species to each other simultaneously. Because of this we started initially by looking at basewise conservation amongst the primates to evaluate potential signatures of evolutionary selection at particular positions of nucleotide bases. The alignment showed that significant conservation (blue bar graph) has been maintained throughout the sequence, as can be seen in Fig. 4. Also seen are sites that are fast evolving (red graph) but these are by far quite few in number and are present in mainly the intronic regions of the sequence. When this alignment was extended to the vertebrates, we found that the conserved residues were now confined mainly to the exons.

When comparing vertebrates, the introns seem to have undergone faster evolution than would be expected under neutral drift (evolutionary change that is caused not by natural selection but due to genetic drift of neutral mutant alleles). A region of around 130 bp in intron 1 appears to have higher conservation than the rest of the sequence. A closer look at this region showed a transcription factor match to ETS1. The *Fugu* sequence was then aligned with just the human sequence to try and find key, ancestral, conserved regions, if any. Pairwise global alignment revealed that less than 10% of the bases in the shorter *Fugu* sequence align with longer human intron 1 sequence (Fig. S4). The alignment improved when the sequences were analysed using local alignment tools, looking for local similarities instead of global alignments (Fig. S5). A similar pattern was observed when the human and mouse intron 1 sequences were aligned (Figs. S6 and S7).

**Figure 5:**
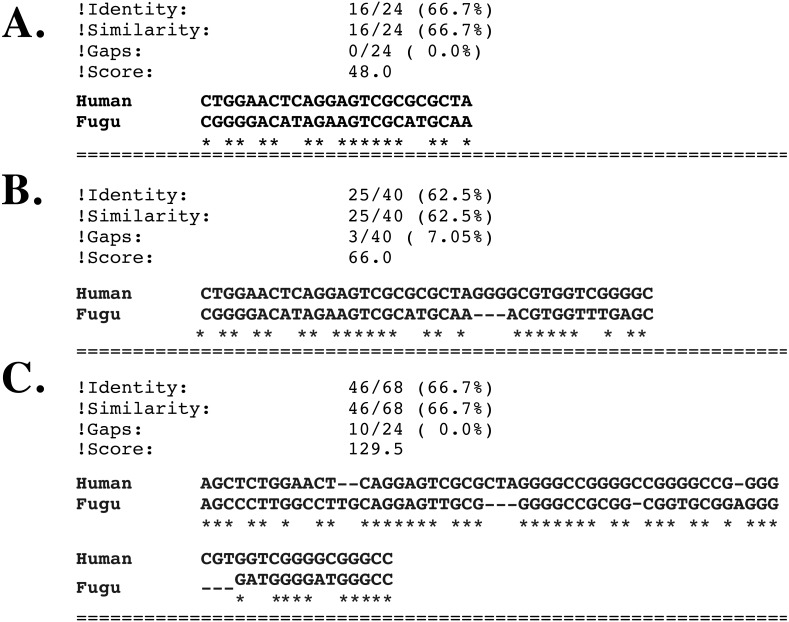
Local alignments of intron 1 sequences. Sequence alignment of Intron 1 sequences using EBI’s EMBOSS matcher (Rice, Longden & Bleasby, 2000) . (A) Hexanucleotide repeat site in human (200 bp upstream of the repeat unis, the repeat sequence themselves, and 200 bp downstream of the repeat units) aligned with the intron 1 sequence of transcript 2 in *Fugu*. (B) The hexanucleotide repeat site in human (same as above but with the repeat units removed) aligned with the intron 1 sequence of transcript 2 in *Fugu*. (C) The hexanucleotide repeat site in human aligned with the intron 1 sequence of transcript 2 in *Fugu*.

Next we looked at the region surrounding the hexanucleotide repeat unit. Human intron 1 sequence was truncated to include only the region surrounding the repeat unit sequence. 200 bases both upstream and downstream of the repeat unit, plus the hexanucleotide sequence itself were used rather than the entire 6,622 bp sequence. The repeat region was then locally aligned to the entire *Fugu* intron sequence (576 bp). This alignment showed that just before the start of the repeat sequence (from position 201) there is a region of 24 nucleotides that has around 67% sequence identity (Fig. 5A). Repetitive motifs/elements are normally masked prior to sequence comparison to prevent spurious alignments. Since the *Fugu* sequence does not contain the repeat expansions, the hexanucleotide repeat units were removed from the human sequence and the two sequences were re-aligned. The removal of the repeat unit resulted in a sequence of 40 nucleotides on either side of the original repeat site with about 63% sequence identity (Fig. 5B). When the shortened version of the human sequence and the mouse intron sequences were locally aligned, we identified a 68 bp region with 67% sequence identity, which spans the repeat site (Fig. 5C).

The hexanucleotide repeat expansions in the non-coding region of *C9orf72* gene are believed to be causal for ALS and FTD. So, we compared the presence/absence of this signature motif in the *C9orf72* gene across species. The *C9orf72* hexanucleotide GGGGCC repeat unit is found 241 bp into the sequence of intron 1 in human transcript variants 2 and 3, but not transcript 1. The intron sequence from transcript 2 is the longest, and was therefore used in our analyses. An unrepeated GGGGCC was also found further downstream in the sequence of human transcript 2 at position 6,245. The GGGGCC sequence is also found in the intron 1 sequence for chimpanzee, human’s closest relative, at position 2,220. However, the repeat unit is only found once and this position is significantly more downstream compared to the human sequence. Surprisingly, the promoter region (before exon 1) in chimpanzee bears three of the hexanucleotide repeat units occurring in tandem. The next closest species to human, gorilla, does not contain the hexanucleotide sequence in intron 1 although it is found in other primates including macaques, orangutans and marmosets. Interestingly, mouse, with the longest intron 1 amongst all the organisms compared, has two GGGGCC repeat units in this region, albeit not in tandem. The GGGGCC sequence was not seen in intron 1 of any of the *Fugu* transcripts.

### Identification of putative immediate upstream elements

In the absence of biological data from experimental methods such as protein-binding microarrays, Chromatin Immunoprecipitation followed by Deep sequencing (ChIp-seq), we took a computational approach to identify the binding sites of DNA-associated proteins using DiAlign TF and MatInspector (Cartharius et al., 2005). To this end, the human *C9orf72* gene up to the end of intron 1 together with 2,000 bp of the 5′-flanking region was aligned with the corresponding murine and *Fugu* sequences. The lack of sequence conservation throughout the alignment resulted in only one TF-binding site (within the aligned regions) that was common to all the three sequences. The transcription factor found common in all the three sequences was ETS1. So, in an attempt to find more transcription factor binding sites that are common to all three sequences, the sequences were scanned by MatInspector without performing any alignment. The matrices used for analysis were filtered based on the tissues they are associated with. The tissues chosen were brain, nervous system, neuroglia, neurons and spinal cord. A core similarity of 1.0 was used for the search. The program found a total of 46 TFs common to all three sequences out of which 18 general regulatory elements were omitted. Only those likely to be preferentially associated with nervous tissue-specific expression were retained (Table 3). The TFs identified play an important role in the development of the nervous system including neuronal differentiation, migration and fate specification (Table 3).

**Table 3:** Transcription factors identified using MatInspector (Cartharius et al., 2005).

Matrix family	*p*-value^∗^	**Description**	**GO terms associated with the matrix family**
V$ETSF	0.00685405	Human and murine ETS1 factors	Motor neuron axon guidance, neuron fate specification, neuron maturation, peripheral nervous system neuron development, positive regulation of neuron death and differentiation.
V$SORY	0.00970298	SOX/SRY-sex/testis determining and related HMG box factors	Brain development, Central, enteric, sympathetic and peripheral nervous system development, CNS neuron differentiation, myelination in peripheral nervous system, spinal cord development, spinal cord motor neuron differentiation and ventral spinal cord interneuron specification.
V$RXRF	0.0124996	RXR heterodimer binding sites	Brain development, central and peripheral nervous system development, CNS neuron differentiation, CNS projection neuron axonogenesis, dopaminergic and midbrain neuron differentiation, forebrain neuron development, neuron differentiation, migration and maturation, negative regulation of neuron apoptotic process, and positive regulation of neuron differentiation.
V$WHNF	0.0208955	Winged helix binding sites	Neuron fate commitment, ventral spinal cord interneuron fate commitment.
V$HOMF	0.0231715	Homeodomain transcription factors	Brain development, Central and enteric nervous system development, neuron differentiation, neuron migration, negative regulation of neuron apoptotic process and regulation of neuron differentiation.
V$BPTF	0.0255628	Bromodomain and PHD domain transcription factors	Brain development.
V$RP58	0.0257514	RP58 (ZFP238) zinc finger protein	Cerebellum development, cerebral cortex development, hippocampus development and neuron development.
V$FKHD	0.0346126	Fork head domain factors	Brain development and morphogenesis, enteric, sympathetic and peripheral nervous system development, CNS neuron development, myelination in central nervous system, regulation of nervous system development, cell morphogenesis in neuron differentiation, dopaminergic neuron differentiation, motor neuron axon guidance, negative regulation of neuron differentiation, neuron development and differentiation, neuron fate determination and specification, neuron stem cell population maintenance, pyramidal neuron migration, positive regulation of neuron apoptotic process and differentiation, spinal cord development and ventral spinal cord interneuron specification.
V$NKXH	0.0376881	NKX homeodomain factors	Brain development, nervous system development, cerebral cortex, forebrain neuron differentiation, spinal cord motor neuron differentiation, motor neuron axon guidance, forebrain neuron fate commitment, negative regulation of neuron apoptotic process, and positive regulation of neuron differentiation, neuron fate commitment, neuron fate specification, neuron migration, spinal cord oligodendrocyte cell differentiation and fate specification, ventral spinal cord interneuron differentiation and fate determination.
V$IRXF	0.0537914	Iroquois homeobox transcription factors	CNS development, CNS neuron differentiation, negative regulation of neuron differentiation, neuron maturation, positive regulation of neuron differentiation and retinal bipolar neuron differentiation.
V$ATBF	0.06225	AT-binding transcription factor	Brain development, regulation of neuron differentiation.
V$ZICF	0.0637499	Members of ZIC-family, zinc finger protein of the cerebellum	Brain development, cell proliferation in forebrain, midbrain and hindbrain, CNS development, determination of right/left asymmetry in nervous system, forebrain morphogenesis, retinal ganglion cell axon guidance, spinal cord development.
V$ABDB	0.0678214	Abdominal-B type homeodomain transcription factors	Peripheral nervous system neuron development and spinal cord motor neuron cell fate specification.
V$SIXF	0.0726783	Sine oculis (SIX) homeodomain factors	Brain development, peripheral nervous system neuron development, generation of neurons, negative regulation of neuron apoptotic process and neuron differentiation, neuron fate specification.
V$BRNF	0.0784254	Brn POU domain factors	Brain development, CNS neuron differentiation, myelination in peripheral nervous system, peripheral nervous system neuron development and differentiation, forebrain neuron differentiation, neuron development, differentiation, neuron fate commitment and fate specification, neuron projection development and negative regulation of neuron apoptotic process.
V$HOXC	0.0805504	HOX-PBX complexes	Brain development and segmentation, CNS neuron differentiation, peripheral nervous system neuron development and differentiation, motor neuron axon guidance, positive & negative regulation of neuron differentiation, neuron development, differentiation and migration, dorsal spinal cord development and spinal cord motor neuron cell fate specification.
V$HBOX	0.086678	Homeobox transcription factors	Brain development, embryonic brain development, autonomic nervous system development, CNS development and neuron differentiation, cell morphogenesis involved in neuron differentiation, dopaminergic neuron differentiation, forebrain neuron development, motor neuron axon guidance, negative regulation of neuron death and neuron apoptotic process, neuron development, differentiation, death, fate commitment, migration, fate specification and projection morphogenesis, sensory neuron axon guidance, subpallium neuron fate commitment, spinal cord association neuron differentiation, spinal cord neuron cell fate specification, spinal cord motor neuron differentiation and spinal cord interneuron specification.
V$LHXF	0.0890478	Lim homeodomain factors	Brain development, CNS neuron development and differentiation, peripheral nervous system neuron axonogenesis and neuron development, GABAergic and dopaminergic neuron differentiation, forebrain neuron development, differentiation and fate commitment, lateral motor column neuron migration, medial motor column neuron differentiation, midbrain-hindbrain boundary development, motor neuron axon guidance, negative regulation of neuron apoptotic process and differentiation, neuron development, differentiation, fate commitment, fate specification, maturation and migration, positive regulation of Wnt-mediated midbrain dopaminergic neuron differentiation, spinal cord development, spinal cord association neuron differentiation, spinal cord motor neuron cell fate specification, spinal cord motor neuron differentiation, ventral spinal cord interneuron specification. and visceral motor neuron differentiation.
V$BRN5	0.0896913	Brn-5 POU domain factors	Brain development, CNS development and ganglion mother cell fate determination.
V$DLXF	0.095084	Distal-less homeodomain transcription factors	Brain and nervous system development, neuron development and differentiation, negative regulation of neuron apoptotic process and regulation of transcription from RNA polymerase II promoter involved in forebrain neuron fate commitment.
V$HIFF	0.0956065	Hypoxia inducible factor, bHLH/PAS protein family	Brain development, CNS development, dopaminergic neuron differentiation, motor neuron axon guidance, negative regulation of oxidative-stress induced neuron intrinsic apoptotic signalling pathway and neuron apoptotic process.
V$TALE	0.0960602	TALE homeodomain class recognizing TG motifs	Brain morphogenesis, enteric nervous system development, neural crest cell migration involved in autonomic nervous system development and positive & negative regulation of neuron differentiation.
V$RORA	0.0967563	v-ERB and RAR-related orphan receptor alpha	Brain development.
V$HESF	0.0988147	Vertebrate homologues of enhancer of split complex	Brain development, CNS development, myelination in CNS, peripheral nervous system development, GABAergic neuron differentiation in basal ganglia, cell morphogenesis involved in neuron differentiation, positive & negative regulation of neuron differentiation, negative regulation of neuron projection development, neuron stem cell population maintenance, regulation of neuronal synaptic plasticity and regulation of timing of neuron differentiation.
V$HOXH	0.0995363	HOX-MEIS1 heterodimers	Enteric nervous system development, peripheral nervous system neuron development, neural crest cell migration involved in autonomic nervous system development, negative regulation of neuron differentiation and spinal cord motor neuron cell fate specification.
V$BCDF	0.103333	Bicoid-like homeodomain transcription factors	Brain development, CNS development, dopaminergic neuron differentiation, positive regulation of neuron apoptotic process, neuron development, neuron differentiation, neuron fate commitment, neuron fate determination and neuron proliferation in midbrain.
V$NKX6	0.105076	NK6 homeobox transcription factors	CNS myelination and neuron differentiation, positive regulation of neuron differentiation, regulation of neuron migration, regulation of transcription from RNA polymerase II promoter involved in spinal cord motor neuron fate specification, spinal cord motor neuron differentiation and regulation of transcription from RNA polymerase II promoter involved in ventral spinal cord interneuron specification.
V$NEUR	0.105134	NeuroD, Beta2, HLH domain	Brain development, CNS development, CNS neuron development, enteric nervous system development, peripheral nervous system development, peripheral nervous system neuron development, sympathetic and parasympathetic nervous systems development, commitment of neuronal cell to specific neuron type in forebrain, dopaminergic neuron differentiation, forebrain neuron development and differentiation, generation of neurons, positive & negative regulation of neuron differentiation, noradrenergic neuron differentiation and neuron fate commitment, neuron development, neuron differentiation, neuron fate commitment, neuron fate determination, neuron migration, regulation of timing of subpallium neuron differentiation, spinal cord association neuron differentiation, spinal cord motor neuron cell fate specification, spinal cord motor neuron differentiation, spinal cord oligodendrocyte cell differentiation and fate specification, subpallium neuron fate commitment, sympathetic ganglion development, spinal reflex action, dorsal spinal cord development, spinal cord development, ventral spinal cord interneuron differentiation and fate commitment, vestibulocochlear nerve development and trigeminal nerve development.

**Figure 6:**
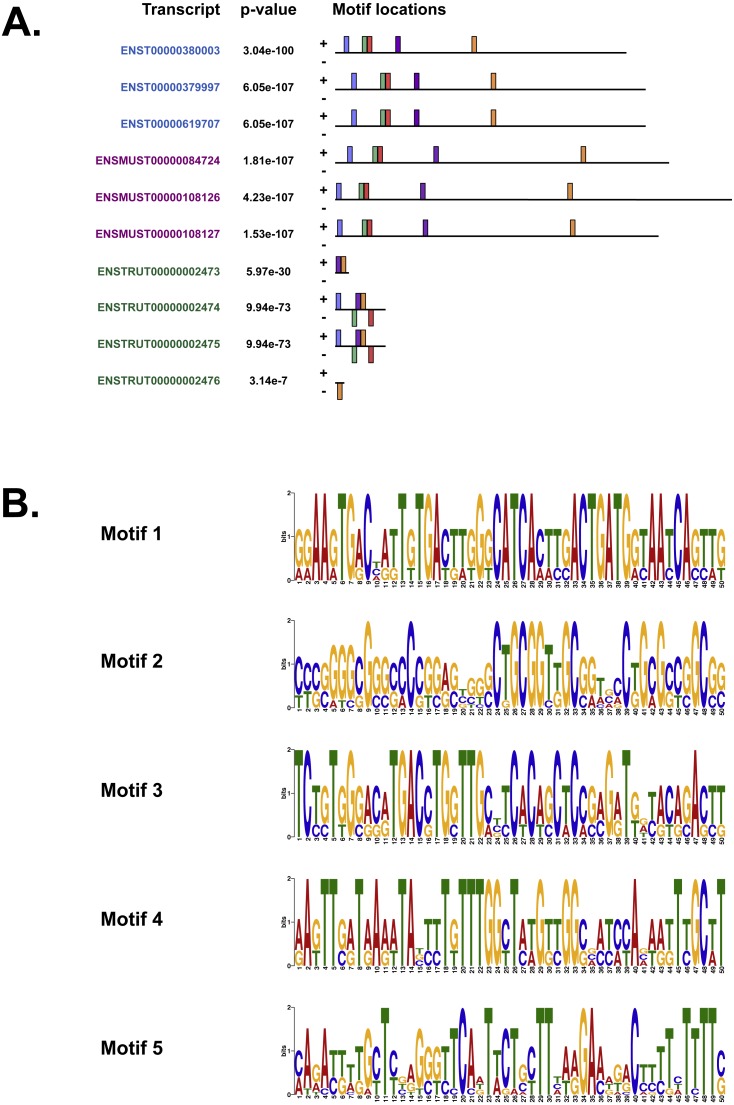
The motifs identified by the MEME suite. (A) The location of the top five motifs found by MEME (Bailey & Elkan, 1994) on the different species. The motifs are coloured differently; motif 1, red; motif 2, blue; motif 3, green; motif 4, yellow and motif 5, purple. (B) The top four motifs (motifs 1, 2, 3 and 4 from above) identified by MEME (Bailey & Elkan, 1994). Motif 5 is not shown because GOMo (Buske et al., 2010) could not predict any biological function that could be associated with the motif.

We also used MEME (Bailey & Elkan, 1994), Tomtom (Gupta et al., 2007) and GOMo (Buske et al., 2010), three motif-based sequence analysis tools from the MEME suite of programs to analyse the upstream regulatory region in human, mouse and *Fugu*. MEME (Bailey & Elkan, 1994) identifies motifs based on the assumption that TF motifs are more likely to be conserved in the regulatory region of a set of orthologous genes. We identified five potential TF binding motifs located at similar positions within the alignment of all the three species (Fig. 6A). The motifs discovered by MEME (Bailey & Elkan, 1994) (Fig. 6B) were searched against databases of known TF motifs using Tomtom (Gupta et al., 2007). TF matrix families identified by Tomtom (Gupta et al., 2007) (Table S2) are consistent with those determined by MatInspector (Cartharius et al., 2005). The MEME motifs were further analysed by the program GOMo (Buske et al., 2010) to determine significant/specific association of these motifs with genes that are linked to one or more gene ontology terms. This approach did not require the use of multiple sequence alignments as each comparative sequence was independently queried for association between a TF and a gene ontology (GO) term, thus avoiding drawbacks such as imperfect alignments or motif drift (Reddy, DeLisi & Shakhnovich, 2005). The GOMo analysis predicted several interesting biological roles for the motifs discovered by MEME. The significant predictions were neuron differentiation, ATP binding, axon guidance and negative regulation of transcription from RNA polymerase II promoter.

### CpG islands and DNA methylation

CpG islands are typically common near transcription start sites and may be associated with promoter regions. These islands are rare in vertebrates because over time the methylated cytosines tend to spontaneously deaminate into thymines. Recent studies have shown that the CpG island, located at the 5′ end of the repeat expansion, is hypermethylated and could potentially lead to reduction of modulation of the disease phenotype (Xi et al., 2013; Belzil et al., 2013). It is believed that hypermethylation is neuroprotective because it inhibits transcription of the mutant *C9orf72* leading to reduced RNA foci and dipeptide aggregate formation (Liu et al., 2014; McMillan et al., 2015; Xi et al., 2015). To this end, we looked at the CpG islands predicted by the UCSC genome browser (Human genome (version hg 38), Mouse genome (version mm 9), *Tetraodon* genome (version tetNig2), and Zebrafish genome (version danRer10)). What was instantly obvious was that only human *C9orf72* gene has a CpG island, located at both 5′ and 3′ end of the region containing the repeat unit (Fig. 4). The 5′ CpG island seen in human intron 1 is absent in mouse, lamprey and zebrafish. To see if there was conservation of the CpG islands in primates in the promoter region, we did a pairwise alignment of the CpG island rich region at the 5′-end of human gene with that of that of the chimpanzee gene. We found that almost all the CpG dinucleotides that were investigated by Xi et al. (2013) are conserved in chimpanzee.

## Discussion

Structural polymorphism of the *C9orf72* hexanucleotide repeat expansion leads to ALS/FTD pathology. Studies up until now have concentrated mainly on the clinical and pathological aspects of the gene. In this study, we systematically extracted and analyzed the genetic level organization and conservation of *C9orf72* by comparative genomic analysis. A comprehensive understanding of the origin, and regulation of the *C9orf72* is essential for our understanding of the function of *C9orf72* protein.

In all the species we compared, *C9orf72* has more than one splice variant except *Tetraodon* and the Stickleback, both of which have only one transcript. It is, however, possible that this differential transcript coverage is a result of incomplete transcriptome analysis in species such as the *Tetraodon* and Stickleback.

The genetic map (order of genes) is ordinarily unique for a given species. However, during the course of evolution it is possible that segments of DNA get rearranged in one species relative to the other resulting in the physical co-localisation of genes. It is becoming increasingly apparent that this conservation or maintenance of syntenic blocks across vertebrate lineages has strong implications in human diseases caused by genetic predisposition (Peltonen & McKusick, 2001). Analysis of synteny between human and mouse *C9orf72* using three different servers showed that the gene order between the two species is well conserved (Fig. 3A). The same gene order is found independent of strand orientation. *MOB3B, IFNκ and LINGO2* stand out because they show very close linkage with *C9orf72* on chromosome 9.

Syntenic regions calculated by Ensembl from pairwise whole genome alignments also revealed the same set of genes on both human and mouse chromosome. However, not all of these homologous genes were present in the chromosome level comparison between human and *Tetraodon*, stickleback and zebrafish (since the Ensembl database lacked an annotated *Fugu* genome, it could not be configured to calculate syntenic regions). Amongst the sub-cluster of genes that co-localise very closely to *C9orf72* physically, the only gene that shows conserved linkage across all species we compared is *LINGO2*. It is conceivable that there could be a link between *C9orf72* and *LINGO2* relating to expression as both are expressed in the neuronal tissues. LINGO2 has been implicated in Parkinson’s disease and essential tremors (a disorder of the nervous system) (Wu et al., 2011).

In addition to LINGO2, 13 other genes identified in the syntenic region are also associated with ALS, FTD or some form of neuronal/nervous disorder like spinocerebellar ataxia and epilepsy (Table S1). All these disease-associated genes map in a contiguous linkage group whilst maintaining the relative order in all the species we compared: human, mouse, *Tetraodon*, stickleback and zebrafish. Interestingly, one of the genes in the syntenic region, *CHMP5* (chromatin-modifying protein 5) is thought to be involved in endocytosis and necroptosis. CHMP5 along with CHMP2B are components of ESCRT-III (endosomal sorting complex required for transport III). Mutations in *CHMP2B* have been shown to cause ALS, FTD (with or without parkinsonism) and presenile dementia (Skibinski et al., 2005; Cannon et al., 2006; Parkinson et al., 2006; Cox et al., 2010). Thus, there seems to be a significant concordance between conserved synteny and the location of orthologous counterparts of human disease genes. Clustering of disease genes in genomic regions of increased syntenic conservation suggests that genome organization is perhaps constrained by gene function. This also helps identify genomes that can be experimentally studied to produce animal models of human diseases.

The strikingly similar architecture observed in the syntenic regions is however not seen when comparing non-coding sequences, in particular the intron 1 sequence. Variation in coding regions of a functionally important gene are less than 1% of the total and are more likely to have a biological impact because they are more likely to change the protein sequence and hence affect the function of the resultant polypeptide. Impact of noncoding variants, on the other hand, is more difficult to predict because variation in non-coding regions of a gene are more abundant and are hence more difficult to assess for functional importance (Nalpathamkalam et al., 2014). Less than 30% of the sequences align when the human and mouse intron 1 sequences were compared (Fig. S6) indicating that proportion of sequence conserved is much below than that expected. The first intron has been found to be the longest of the introns in the species analyzed. The greater the intron length, the lower the GC content (Gazave et al., 2007), resulting in decreased recombination (Carvalho & Clark, 1999). This suggests the intron is not under the same selection pressure as regions that recombine frequently. This decreased selection pressure could explain why the sequence in this region is so variable.

Another characteristic feature was the presence/absence of the hexanucleotide sequence itself. Our analysis showed that only human intron 1 harbors the hexanucleotide sequence. Adding to this variability is the lack of a CpG island in the 5′-region of vertebrate *C9orf72* except in human and the chimpanzee. CpG islands are believed to be an epigenetic mechanism that adds another layer of gene regulation. It is possible that the CpG islands at the 5′-end of the *C9orf72* gene in human and the chimpanzee, which also harbors the hexanucleotide repeat unit in its promoter region in human intron 1, might have evolved as a compensatory adaptation to regulate gene expression or to mask the deleterious effect of the hexanucleotide repeat units.

Regulation of gene expression and cellular processes is achieved by the binding of transcription factor proteins (TFs) to specific conserved DNA sequences. Functional studies provide insights into the type or number of genes regulated by a single TF. However, in order to understand regulation by a group of TFs acting in a coordinated fashion, characterization of the upstream regulatory regions of a gene for TF binding sites becomes essential. Recent studies have mainly focused on understanding the epigenetic mechanisms that may be involved in regulating the transcriptional silencing of *C9orf72* in repeat expansion disease pathology (Xi et al., 2013; Xi et al., 2015).

Rizzu et al. (2016) studied the landscape surrounding the *C9orf72* locus using global CAGEseq expression data to understand regulation of *C9orf72* expression. They found that the architecture of the region governing transcription at the *C9orf72* locus was complex. They identified new transcription start sites (TSSs) on both sense and antisense strands that could potentially lead to novel *C9orf72* transcripts. These transcripts (annotated and non-annotated) were shown to express differentially in patients with ALS. The study suggests that the regulation of *C9orf72* transcription is ill understood.

Our analysis of the upstream regulatory region in human, mouse and *Fugu* using MatInspector (Cartharius et al., 2005) identified several conserved TF binding sites which have relevance for CNS-specific expression of *C9orf72* (Fig. S1). Similar to MatInspector, the MEME suite of programs, used for regulatory sequence analysis, also identified TF binding motifs that associate with genes involved in regulation of neuronal differentiation and axon guidance (Fig. 6).

In addition to TFs that regulate genes controlling neuronal differentiation and axon guidance, analysis of the upstream regulatory region by MEME (Bailey & Elkan, 1994) identified TF binding motifs which are predicted by GOMo (Buske et al., 2010) to associate with genes that usually regulate the innate immune system and GTPase activity (Table S2).

Association of motif 1 (Fig. 6B) with genes regulating innate immune system is in agreement with the findings of Rizzu et al. (2016) which show *C9orf72* expression in myeloid cells and a seven-fold higher expression of *C9orf72* in CD14+ monocytes after exposure to microbes. The GO terms significantly associated with Motif 2 is GTPase activity. Amongst the several genes associated with this molecular function, one that stands out is RRAGC (Ras-related GTP-binding protein C); which is involved in activation of the mTOR signalling cascade. Apart from its role as a Rab-GEF, the tri-molecular complex of *C9orf72* with SMCR8 (Amick, Roczniak-Ferguson & Ferguson, 2016) and WDR41 (Sullivan et al., 2016) regulates signal transduction via mTORC1 complex.

From our analysis it can be seen that the TF binding motifs predicted to regulate the expression *C9orf72* genes are important in key cellular/biological processes in the nervous system. The information obtained from such a comparative genomics approach will facilitate the design of future functional studies in terms of gene function and regulation of *C9orf72*.

## Conclusions

ALS and FTD, two late onset neurodegenerative diseases, have been shown to share overlapping cellular pathologies and genetic origins. Although it is clear that mutations in one of several different genes can cause/are risk factors for ALS the overriding question that remains as yet unanswered is: How? The genetic link between *C9orf72* gene and the ALS-FTD spectrum points to its putative role in lysosome biogenesis, vesicular trafficking, autophagy and mTORC1 signalling. Advances in the understanding and treatment of diseases depend heavily on our ability to pinpoint specific genetic/cellular/environmental factors that underlie disease manifestation. Our study presented here analyses the available data for *C9orf72* at the gene level and provides some insights into the regulation of *C9orf72* and its close linkage to other genes implicated in nervous system disorders. Our report provides a previously unavailable systematic perspective on *C9orf72* for streamlining future research efforts.

##  Supplemental Information

10.7717/peerj.4391/supp-1 Table S1 Genes located in the region of conserved synteny between human and mouse (Catchen, Conery & Postlethwait, 2009)

10.7717/peerj.4391/supp-2 Table S2 Transcription factors and GO terms associated with MEME motifs (Bailey & Elkan, 1994) as identified by Tomtom (Gupta et al., 2007) and GOMo (Buske et al., 2010)

10.7717/peerj.4391/supp-3 Figure S1 Phylogenetic analysis of ANG (angiogenin) A rooted phylogenetic tree of all the species that express ANG generated by the Ensemble Compara server. The tree was drawn using TreeDyn (Chevenet et al., 2006). The branch-length scale represents substitutions per base pair.

10.7717/peerj.4391/supp-4 Figure S2 Phylogenetic analysis of IFNK (Interferon Kappa) A rooted phylogenetic tree of all the species that express IFN *κ* generated by the Ensemble Compara server. The tree was drawn using TreeDyn (Chevenet et al., 2006). The branch-length scale represents substitutions per base pair.

10.7717/peerj.4391/supp-5 Figure S3 Phylogenetic analysis of NDUFB6 (NADH:Ubiquinone Oxidoreductase Subunit B6) A rooted phylogenetic tree of all the species that express NDUFB6 generated by the Ensemble Compara server. The tree was drawn using TreeDyn (Chevenet et al., 2006). The branch-length scale represents substitutions per base pair.

10.7717/peerj.4391/supp-6 Figure S4 Global alignment of intron 1 sequence from human and Fugu Sequence alignment of Intron 1 sequences using EBI’s EMBOSS Needle (Rice, Longden & Bleasby, 2000).

10.7717/peerj.4391/supp-7 Figure S5 Local alignment of intron 1 sequence from human and Fugu Sequence alignment of Intron 1 sequences using EBI’s EMBOSS Matcher (Rice, Longden & Bleasby, 2000).

10.7717/peerj.4391/supp-8 Figure S6 Global alignment of intron 1 sequence from human and mouse Sequence alignment of Intron 1 sequences using EBI’s EMBOSS Needle (Rice, Longden & Bleasby, 2000).

10.7717/peerj.4391/supp-9 Figure S7 Local alignment of intron 1 sequence from human and mouse Sequence alignment of Intron 1 sequences using EBI’s EMBOSS Needle (Rice, Longden & Bleasby, 2000).

10.7717/peerj.4391/supp-10 Figure S8 Transcription factor binding sites common to human, mouse and Fugu sequences Sequences, represented by black lines, are vertically aligned along their start positions. Each matrix family is represented by one unique colour wherein matches to matrices of the same family are painted the same colour. Matches found on the positive or negative strand are shown on top or below the sequence line, respectively. Transcription factors were identified by the program MatInspector (Cartharius et al., 2005).

10.7717/peerj.4391/supp-11 Supplemental Information 1 Transcription factors identified using MatInspector Raw result for Common TF search using a core similarity of 0.75 and all tissues.

10.7717/peerj.4391/supp-12 Supplemental Information 2 Transcription factors identified using MatInspector Raw result for Common TF search using a core similarity of 0.75 and only selected tissues.

10.7717/peerj.4391/supp-13 Supplemental Information 3 Transcription factors identified using MatInspector Raw result for Common TF search using a core similarity of 1.0 and only selected tissues.

## Additional Information and Declarations

### Data Availability

The following information was supplied regarding data availability: The research in this article is based on bioinformatics analysis. As such, we used sequences that are freely available in the public domain (http://www.ensembl.org). The results of our Common TF analysis are included in the Supplemental Files.
