# Supplementary material for: A comparative bioinformatic analysis of C9orf72
Source: PeerJ. 2018 Feb 19;6:e4391. doi: 10.7717/peerj.4391 (PMC5822839; doi:10.7717/peerj.4391)
Supplement: Table S1 [file peerj-06-4391-s001.docx]

|  | **Gene name** | **Function/disorder** |
| --- | --- | --- |
| 1 | **MOB3B** | - |
| 2 | **IFNK** | Associated with IFNK include FTD and ALS |
| 3 | **C9orf72** | Associated with FTD and ALS |
| 4 | **LINGO2** | Associated with essential tremor and Parkinson disease |
| 5 | **ACO1** | Associated with Friedreich spinocerebellar ataxia |
| 6 | **DDX58** | Associated with Singleton-Merten syndrome 2 and Singleton-Merten dysplasia |
| 7 | **TOPORS** | Associated with retinitis pigmentosa |
| 8 | **NDUFB6** | Associated with inclusion body myopathy with early-onset Paget disease of bone and/or Frontotemporal Dementia |
| 9 | **TMEM215** | - |
| 10 | **APTX** | Associated with autosomal recessive spinocerebellar ataxia |
| 11 | **DNAJA1** | - |
| 12 | **SMU1** | - |
| 13 | **B4GALT1** | - |
| 14 | **SPINK4** | - |
| 15 | **BAG1** | - |
| 16 | **CHMP5** | Involved in endocytosis and necroptosis |
| 17 | **NFX1** | - |
| 18 | **AQP7** | - |
| 19 | **AQP3** | - |
| 20 | **NOL6** | - |
| 21 | **UBE2R2** | - |
| 22 | **UBAP2** | - |
| 23 | **DCAF12** | - |
| 24 | **UBAP1** | - |
| 25 | **KIF24** | - |
| 26 | **NUDT2** | - |
| 27 | **KIAA1161** | - |
| 28 | **C9orf24** | - |
| 29 | **FAM219A** | - |
| 30 | **DNAI1** | Ciliary dyskinesia |
| 31 | **ENHO** | - |
| 32 | **CNTFR** | ALS, attention-deficit hyperactivity disorder |
| 33 | **RPP25L** | - |
| 34 | **DCTN3** | - |
| 35 | **ARID3C** | - |
| 36 | **SIGMAR1** | ALS |
| 37 | **GALT** | - |
| 38 | **IL11RA** | familial temporal lobe epilepsy 2 |
| 39 | **CCL27** | - |
| - Indicates genes where disorders have not been identified as yet | | |
