# Supplementary material for: A comparative bioinformatic analysis of C9orf72
Source: PeerJ. 2018 Feb 19;6:e4391. doi: 10.7717/peerj.4391 (PMC5822839; doi:10.7717/peerj.4391)
Supplement: Table S2 [file peerj-06-4391-s002.docx]

| **MEMEMOTIF** | **NO. OF MATCHES** | **TRANSCRIPTION FACTOR FAMILIES** | **TOP SPECIFIC PREDICTIONS ASSOCIATED WITH THE MOTIF** |
| --- | --- | --- | --- |
| 1 | 25 | [LHX6_full_3](http://meme-suite.org/opal-jobs/appTOMTOM_4.12.01516189468806-2115812527/tomtom.html#match_0_11),  [Lhx8_DBD_3](http://meme-suite.org/opal-jobs/appTOMTOM_4.12.01516189468806-2115812527/tomtom.html#match_0_12),  [MA0070.1 (PBX1)](http://meme-suite.org/opal-jobs/appTOMTOM_4.12.01516189468806-2115812527/tomtom.html#match_0_37),  [SREBF2_DBD](http://meme-suite.org/opal-jobs/appTOMTOM_4.12.01516189468806-2115812527/tomtom.html#match_0_25),  [TFE3_DBD](http://meme-suite.org/opal-jobs/appTOMTOM_4.12.01516189468806-2115812527/tomtom.html#match_0_30),  [MA0828.1 (SREBF2(var.2))](http://meme-suite.org/opal-jobs/appTOMTOM_4.12.01516189468806-2115812527/tomtom.html#match_0_68),  [Srebf1_DBD](http://meme-suite.org/opal-jobs/appTOMTOM_4.12.01516189468806-2115812527/tomtom.html#match_0_26),  [TFEB_full](http://meme-suite.org/opal-jobs/appTOMTOM_4.12.01516189468806-2115812527/tomtom.html#match_0_31),  [MA0692.1 (TFEB)](http://meme-suite.org/opal-jobs/appTOMTOM_4.12.01516189468806-2115812527/tomtom.html#match_0_63),  [MA0829.1 (Srebf1(var.2))](http://meme-suite.org/opal-jobs/appTOMTOM_4.12.01516189468806-2115812527/tomtom.html#match_0_69),  [TFEC_DBD](http://meme-suite.org/opal-jobs/appTOMTOM_4.12.01516189468806-2115812527/tomtom.html#match_0_32),  [MA0871.1 (TFEC)](http://meme-suite.org/opal-jobs/appTOMTOM_4.12.01516189468806-2115812527/tomtom.html#match_0_70),  [MA0594.1 (Hoxa9)](http://meme-suite.org/opal-jobs/appTOMTOM_4.12.01516189468806-2115812527/tomtom.html#match_0_53),  [MLXIPL_full](http://meme-suite.org/opal-jobs/appTOMTOM_4.12.01516189468806-2115812527/tomtom.html#match_0_13),  [MLX_full](http://meme-suite.org/opal-jobs/appTOMTOM_4.12.01516189468806-2115812527/tomtom.html#match_0_14),  [MA0663.1 (MLX)](http://meme-suite.org/opal-jobs/appTOMTOM_4.12.01516189468806-2115812527/tomtom.html#match_0_61),  [MA0664.1 (MLXIPL)](http://meme-suite.org/opal-jobs/appTOMTOM_4.12.01516189468806-2115812527/tomtom.html#match_0_62),  [USF1_DBD](http://meme-suite.org/opal-jobs/appTOMTOM_4.12.01516189468806-2115812527/tomtom.html#match_0_33),  [BHLHE41_full](http://meme-suite.org/opal-jobs/appTOMTOM_4.12.01516189468806-2115812527/tomtom.html#match_0_1),  [MA0636.1 (BHLHE41)](http://meme-suite.org/opal-jobs/appTOMTOM_4.12.01516189468806-2115812527/tomtom.html#match_0_57),  [UP00012_2 (Bbx_secondary)](http://meme-suite.org/opal-jobs/appTOMTOM_4.12.01516189468806-2115812527/tomtom.html#match_0_82),  [BHLHB2_DBD](http://meme-suite.org/opal-jobs/appTOMTOM_4.12.01516189468806-2115812527/tomtom.html#match_0_0),  [Mlx_DBD](http://meme-suite.org/opal-jobs/appTOMTOM_4.12.01516189468806-2115812527/tomtom.html#match_0_15),  [MA0464.2 (BHLHE40)](http://meme-suite.org/opal-jobs/appTOMTOM_4.12.01516189468806-2115812527/tomtom.html#match_0_47),  [UP00219_2 (Cutl1_3494.2)](http://meme-suite.org/opal-jobs/appTOMTOM_4.12.01516189468806-2115812527/tomtom.html#match_0_90) | olfactory receptor activity, sensory perception of smell, G-protein coupled receptor protein signalling pathway, innate immune response, defense response to bacterium |
| 2 | 38 | [MA0079.3 (SP1)](http://meme-suite.org/opal-jobs/appTOMTOM_4.12.01516189468806-2115812527/tomtom.html#match_1_41),  [MA0146.2 (Zfx)](http://meme-suite.org/opal-jobs/appTOMTOM_4.12.01516189468806-2115812527/tomtom.html#match_1_45),  [UP00088_1 (Plagl1_primary)](http://meme-suite.org/opal-jobs/appTOMTOM_4.12.01516189468806-2115812527/tomtom.html#match_1_88),  [MA0516.1 (SP2)](http://meme-suite.org/opal-jobs/appTOMTOM_4.12.01516189468806-2115812527/tomtom.html#match_1_51),  [MA0506.1 (NRF1)](http://meme-suite.org/opal-jobs/appTOMTOM_4.12.01516189468806-2115812527/tomtom.html#match_1_50),  [MA0750.2 (ZBTB7A)](http://meme-suite.org/opal-jobs/appTOMTOM_4.12.01516189468806-2115812527/tomtom.html#match_1_67),  [KLF16_DBD](http://meme-suite.org/opal-jobs/appTOMTOM_4.12.01516189468806-2115812527/tomtom.html#match_1_10),  [MA0741.1 (KLF16)](http://meme-suite.org/opal-jobs/appTOMTOM_4.12.01516189468806-2115812527/tomtom.html#match_1_65),  [MA0599.1 (KLF5)](http://meme-suite.org/opal-jobs/appTOMTOM_4.12.01516189468806-2115812527/tomtom.html#match_1_54),  [MA1122.1 (TFDP1)](http://meme-suite.org/opal-jobs/appTOMTOM_4.12.01516189468806-2115812527/tomtom.html#match_1_75),  [HINFP1_full_2](http://meme-suite.org/opal-jobs/appTOMTOM_4.12.01516189468806-2115812527/tomtom.html#match_1_3),  [UP00102_1 (Zic1_primary)](http://meme-suite.org/opal-jobs/appTOMTOM_4.12.01516189468806-2115812527/tomtom.html#match_1_89),  [UP00009_2 (Nr2f2_secondary)](http://meme-suite.org/opal-jobs/appTOMTOM_4.12.01516189468806-2115812527/tomtom.html#match_1_81),  [UP00021_1 (Zfp281_primary)](http://meme-suite.org/opal-jobs/appTOMTOM_4.12.01516189468806-2115812527/tomtom.html#match_1_83),  [SP1_DBD](http://meme-suite.org/opal-jobs/appTOMTOM_4.12.01516189468806-2115812527/tomtom.html#match_1_23),  [IRF5_full_1](http://meme-suite.org/opal-jobs/appTOMTOM_4.12.01516189468806-2115812527/tomtom.html#match_1_6),  [MA1420.1 (IRF5)](http://meme-suite.org/opal-jobs/appTOMTOM_4.12.01516189468806-2115812527/tomtom.html#match_1_78),  [MA0116.1 (Znf423)](http://meme-suite.org/opal-jobs/appTOMTOM_4.12.01516189468806-2115812527/tomtom.html#match_1_43),  [IRF8_full](http://meme-suite.org/opal-jobs/appTOMTOM_4.12.01516189468806-2115812527/tomtom.html#match_1_7),  [MA0652.1 (IRF8)](http://meme-suite.org/opal-jobs/appTOMTOM_4.12.01516189468806-2115812527/tomtom.html#match_1_59),  [UP00006_1 (Zic3_primary)](http://meme-suite.org/opal-jobs/appTOMTOM_4.12.01516189468806-2115812527/tomtom.html#match_1_80),  [ZBTB7A_DBD](http://meme-suite.org/opal-jobs/appTOMTOM_4.12.01516189468806-2115812527/tomtom.html#match_1_34),  [MA1108.1 (MXI1)](http://meme-suite.org/opal-jobs/appTOMTOM_4.12.01516189468806-2115812527/tomtom.html#match_1_73),  [IRF4_full](http://meme-suite.org/opal-jobs/appTOMTOM_4.12.01516189468806-2115812527/tomtom.html#match_1_5),  [MA1419.1 (IRF4)](http://meme-suite.org/opal-jobs/appTOMTOM_4.12.01516189468806-2115812527/tomtom.html#match_1_77),  [UP00057_1 (Zic2_primary)](http://meme-suite.org/opal-jobs/appTOMTOM_4.12.01516189468806-2115812527/tomtom.html#match_1_84),  [SP3_DBD](http://meme-suite.org/opal-jobs/appTOMTOM_4.12.01516189468806-2115812527/tomtom.html#match_1_24),  [MA0746.1 (SP3)](http://meme-suite.org/opal-jobs/appTOMTOM_4.12.01516189468806-2115812527/tomtom.html#match_1_66),  [MA0163.1 (PLAG1)](http://meme-suite.org/opal-jobs/appTOMTOM_4.12.01516189468806-2115812527/tomtom.html#match_1_46),  [MA1100.1 (ASCL1)](http://meme-suite.org/opal-jobs/appTOMTOM_4.12.01516189468806-2115812527/tomtom.html#match_1_71),  [MA1102.1 (CTCFL)](http://meme-suite.org/opal-jobs/appTOMTOM_4.12.01516189468806-2115812527/tomtom.html#match_1_72),  [MA0139.1 (CTCF)](http://meme-suite.org/opal-jobs/appTOMTOM_4.12.01516189468806-2115812527/tomtom.html#match_1_44),  [MA0073.1 (RREB1)](http://meme-suite.org/opal-jobs/appTOMTOM_4.12.01516189468806-2115812527/tomtom.html#match_1_39),  [IRF9_full](http://meme-suite.org/opal-jobs/appTOMTOM_4.12.01516189468806-2115812527/tomtom.html#match_1_8),  [MA0653.1 (IRF9)](http://meme-suite.org/opal-jobs/appTOMTOM_4.12.01516189468806-2115812527/tomtom.html#match_1_60),  [TFAP2A_DBD_2](http://meme-suite.org/opal-jobs/appTOMTOM_4.12.01516189468806-2115812527/tomtom.html#match_1_29),  [MA0003.3 (TFAP2A)](http://meme-suite.org/opal-jobs/appTOMTOM_4.12.01516189468806-2115812527/tomtom.html#match_1_35),  [UP00002_1 (Sp4_primary)](http://meme-suite.org/opal-jobs/appTOMTOM_4.12.01516189468806-2115812527/tomtom.html#match_1_79) | ATP binding, transcription factor complex zinc and magnesium ion binding, GTPase activity, negative regulation of transcription from RNA polymerase II promoter |
| 3 | 20 | [Rxra_DBD_2](http://meme-suite.org/opal-jobs/appTOMTOM_4.12.01516189468806-2115812527/tomtom.html#match_2_22),  [RXRG_full_2](http://meme-suite.org/opal-jobs/appTOMTOM_4.12.01516189468806-2115812527/tomtom.html#match_2_21),  [MA0071.1 (RORA)](http://meme-suite.org/opal-jobs/appTOMTOM_4.12.01516189468806-2115812527/tomtom.html#match_2_38),  [MA0089.1 (MAFG::NFE2L1)](http://meme-suite.org/opal-jobs/appTOMTOM_4.12.01516189468806-2115812527/tomtom.html#match_2_42),  [RARG_full_1](http://meme-suite.org/opal-jobs/appTOMTOM_4.12.01516189468806-2115812527/tomtom.html#match_2_17),  [MA1150.1 (RORB)](http://meme-suite.org/opal-jobs/appTOMTOM_4.12.01516189468806-2115812527/tomtom.html#match_2_76),  [MA0526.2 (USF2)](http://meme-suite.org/opal-jobs/appTOMTOM_4.12.01516189468806-2115812527/tomtom.html#match_2_52),  [RXRA_DBD_2](http://meme-suite.org/opal-jobs/appTOMTOM_4.12.01516189468806-2115812527/tomtom.html#match_2_18),  [RXRA_full_2](http://meme-suite.org/opal-jobs/appTOMTOM_4.12.01516189468806-2115812527/tomtom.html#match_2_19),  [RXRG_DBD_2](http://meme-suite.org/opal-jobs/appTOMTOM_4.12.01516189468806-2115812527/tomtom.html#match_2_20),  [TBX21_DBD_3](http://meme-suite.org/opal-jobs/appTOMTOM_4.12.01516189468806-2115812527/tomtom.html#match_2_28),  [IRX2_DBD](http://meme-suite.org/opal-jobs/appTOMTOM_4.12.01516189468806-2115812527/tomtom.html#match_2_9),  [UP00079_1 (Esrra_primary)](http://meme-suite.org/opal-jobs/appTOMTOM_4.12.01516189468806-2115812527/tomtom.html#match_2_87),  [ESRRG_full_3](http://meme-suite.org/opal-jobs/appTOMTOM_4.12.01516189468806-2115812527/tomtom.html#match_2_2),  [MA0643.1 (Esrrg)](http://meme-suite.org/opal-jobs/appTOMTOM_4.12.01516189468806-2115812527/tomtom.html#match_2_58),  [MA0603.1 (Arntl)](http://meme-suite.org/opal-jobs/appTOMTOM_4.12.01516189468806-2115812527/tomtom.html#match_2_55),  [MA0074.1 (RXRA::VDR)](http://meme-suite.org/opal-jobs/appTOMTOM_4.12.01516189468806-2115812527/tomtom.html#match_2_40),  [Nr2f6_DBD_1](http://meme-suite.org/opal-jobs/appTOMTOM_4.12.01516189468806-2115812527/tomtom.html#match_2_16),  [MA0728.1 (Nr2f6(var.2))](http://meme-suite.org/opal-jobs/appTOMTOM_4.12.01516189468806-2115812527/tomtom.html#match_2_64),  [TBX1_DBD_4](http://meme-suite.org/opal-jobs/appTOMTOM_4.12.01516189468806-2115812527/tomtom.html#match_2_27) | protein targeting to mitochondrion, organ morphogenesis, Cell development, cytoplasm, protein binding |
| 4 | 6 | [MA0060.3 (NFYA)](http://meme-suite.org/opal-jobs/appTOMTOM_4.12.01516189468806-2115812527/tomtom.html#match_3_36),  [MA0611.1 (Dux)](http://meme-suite.org/opal-jobs/appTOMTOM_4.12.01516189468806-2115812527/tomtom.html#match_3_56),  [UP00061_2 (Foxl1_secondary)](http://meme-suite.org/opal-jobs/appTOMTOM_4.12.01516189468806-2115812527/tomtom.html#match_3_85),  [UP00235_1 (Hoxc11_3718.2)](http://meme-suite.org/opal-jobs/appTOMTOM_4.12.01516189468806-2115812527/tomtom.html#match_3_91),  [HOXC11_full_2](http://meme-suite.org/opal-jobs/appTOMTOM_4.12.01516189468806-2115812527/tomtom.html#match_3_4),  [MA0502.1 (NFYB)](http://meme-suite.org/opal-jobs/appTOMTOM_4.12.01516189468806-2115812527/tomtom.html#match_3_49) | olfactory receptor activity, sensory perception of smell, G-protein coupled receptor protein signalling pathway, detection of biotic stimulus, defense response to bacterium |
| 5 | 3 | [MA0494.1 (Nr1h3::Rxra)](http://meme-suite.org/opal-jobs/appTOMTOM_4.12.01516189468806-2115812527/tomtom.html#match_4_48),  [UP00078_1 (Arid3a_primary)](http://meme-suite.org/opal-jobs/appTOMTOM_4.12.01516189468806-2115812527/tomtom.html#match_4_86),  [MA1119.1 (SIX2)](http://meme-suite.org/opal-jobs/appTOMTOM_4.12.01516189468806-2115812527/tomtom.html#match_4_74) | olfactory receptor activity, sensory perception of smell, G-protein coupled receptor protein signalling pathway, immune response, regulation of immune response |
