# Supplementary figures and images for: A comparative bioinformatic analysis of C9orf72
Source: PeerJ. 2018 Feb 19;6:e4391. doi: 10.7717/peerj.4391 (PMC5822839; doi:10.7717/peerj.4391)

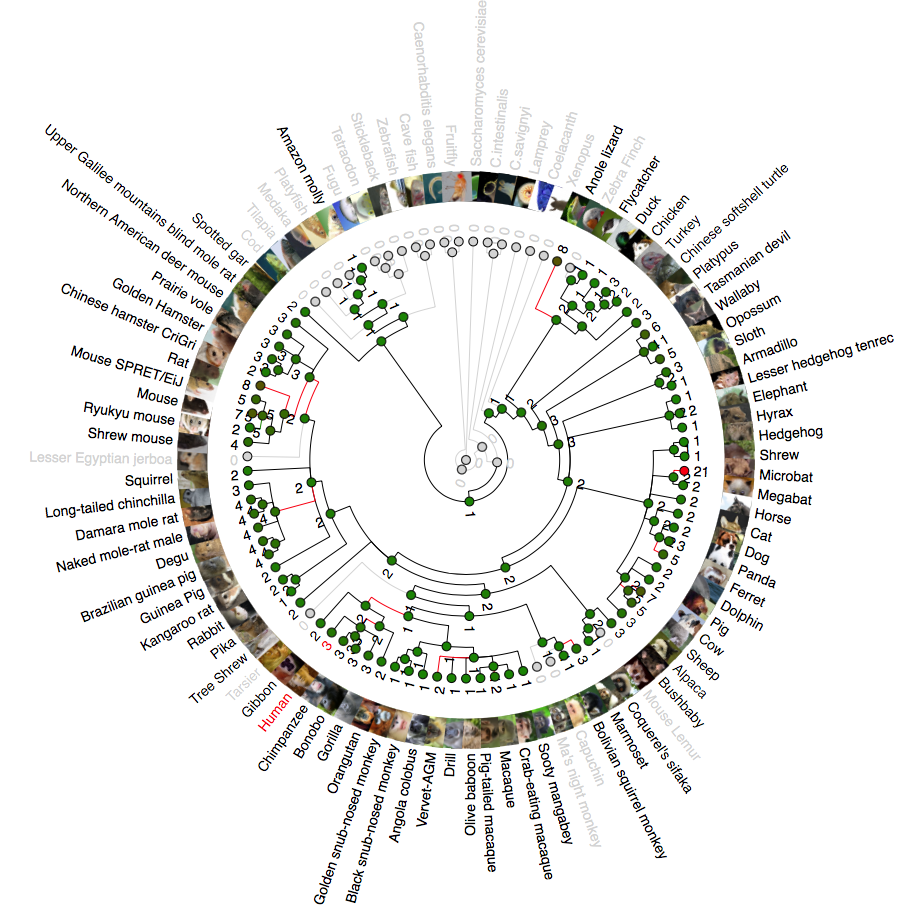

Supplement: Figure S1 — A rooted phylogenetic tree of all the species that express ANG generated by the Ensemble Compara server. The tree was drawn using TreeDyn (Chevenet et al., 2006). The branch-length scale represents substitutions per base pair. [file peerj-06-4391-s003.png]

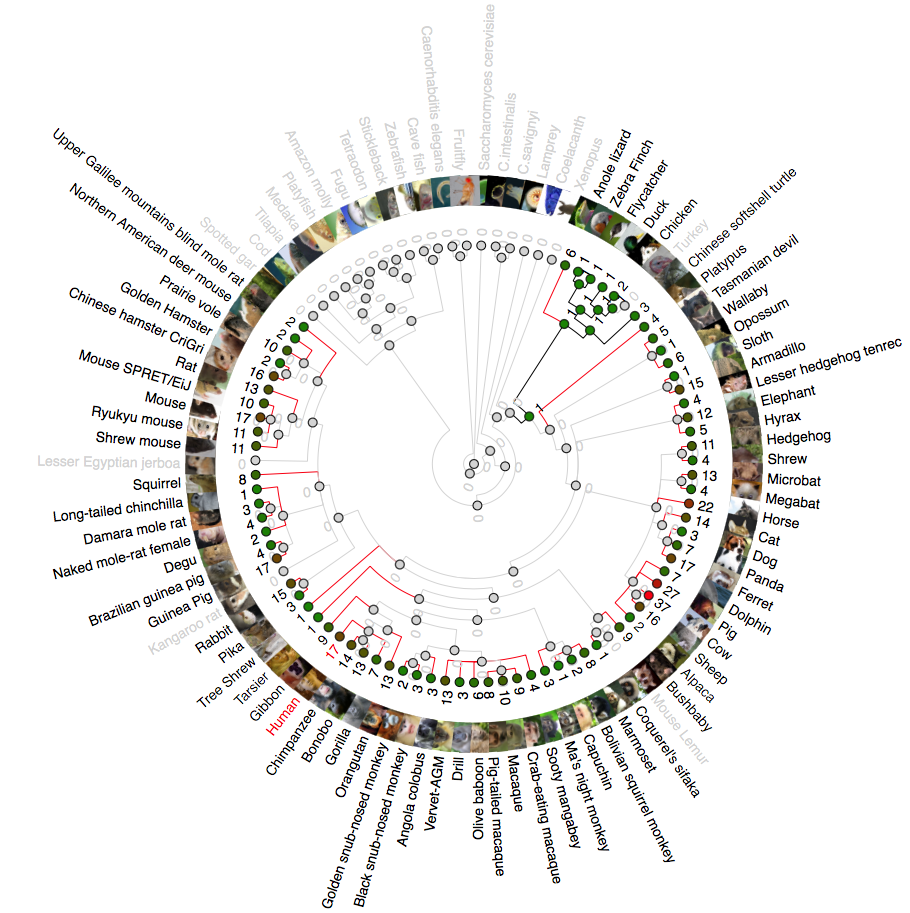

Supplement: Figure S2 — A rooted phylogenetic tree of all the species that express IFN κ generated by the Ensemble Compara server. The tree was drawn using TreeDyn (Chevenet et al., 2006). The branch-length scale represents substitutions per base pair. [file peerj-06-4391-s004.png]

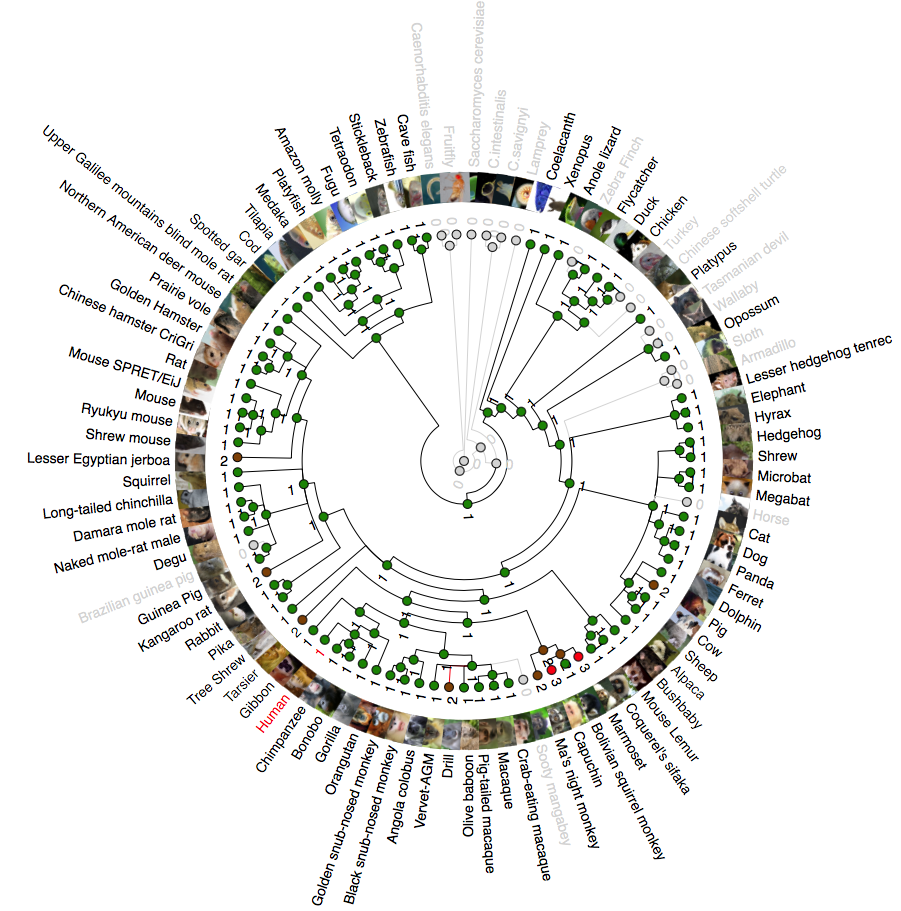

Supplement: Figure S3 — A rooted phylogenetic tree of all the species that express NDUFB6 generated by the Ensemble Compara server. The tree was drawn using TreeDyn (Chevenet et al., 2006). The branch-length scale represents substitutions per base pair. [file peerj-06-4391-s005.png]
