# Supplementary material for: A comparative bioinformatic analysis of C9orf72
Source: PeerJ. 2018 Feb 19;6:e4391. doi: 10.7717/peerj.4391 (PMC5822839; doi:10.7717/peerj.4391)
Supplement: Figure S4 — Sequence alignment of Intron 1 sequences using EBI’s EMBOSS Needle (Rice, Longden & Bleasby, 2000). [file peerj-06-4391-s006.pdf]

```
<html><head></head><body><pre style="word-wrap: break-word; white-space: pre-wrap;">########################################
# Program: needle
# Rundate: Wed 24 Jan 2018 13:29:59
# Commandline: needle
#    -auto
#    -stdout
#    -asequence emboss_needle-I20180124-132957-0052-68239585-p1m.asequence
#    -bsequence emboss_needle-I20180124-132957-0052-68239585-p1m.bsequence
#    -datafile EDNAFULL
#    -gapopen 10.0
#    -gapextend 0.5
#    -endopen 10.0
#    -endextend 0.5
#    -aformat3 pair
#    -snucleotide1
#    -snucleotide2
# Align_format: pair
# Report_file: stdout
########################################

#=======================================
#
# Aligned_sequences: 2
# 1: human
# 2: fugu
# Matrix: EDNAFULL
# Gap_penalty: 10.0
# Extend_penalty: 0.5
#
# Length: 6675
# Identity:     419/6675 ( 6.3%)
# Similarity:   419/6675 ( 6.3%)
# Gaps:        6152/6675 (92.2%)
# Score: 656.5
#
#
#=======================================

human              1 gtgtgtcagccgtccctgctgcccggttgcttctcttttgggggcggggt     50

fugu               1 --------------------------------------------------      0

human             51 ctagcaagagcaggtgtgggtttaggaggtgtgtgtttttgtttttccca    100

fugu               1 --------------------------------------------------      0

human            101 ccctctctccccactacttgctctcacagtactcgctgagggtgaacaag    150

fugu               1 --------------------------------------------------      0

human            151 aaaagacctgataaagattaaccagaagaaaacaaggagggaaacaaccg    200

fugu               1 --------------------------------------------------      0

human            201 cagcctgtagcaagctctggaactcaggagtcgcgcgctaggggccgggg    250

fugu               1 --------------------------------------------------      0

human            251 ccggggccggggcgtggtcggggcgggcccgggggcgggcccggggcggg    300

fugu               1 --------------------------------------------------      0

human            301 gctgcggttgcggtgcctgcgcccgcggcggcggaggcgcaggcggtggc    350

fugu               1 --------------------------------------------------      0

human            351 gagtgggtgagtgaggaggcggcatcctggcgggtggctgtttggggttc    400


fugu               1 --------------------------------------------------      0

human            401 ggctgccgggaagaggcgcgggtagaagcgggggctctcctcagagctcg    450

fugu               1 --------------------------------------------------      0
```

```
human             451 acgcattttttactttccctctcatttctctgaccgaagctgggtgtcggg    500

fugu                1 --------------------------------------------------      0

human             501 ctttcgcctctagcgactggtggaattgcctgcatccgggccccgggctt    550

fugu                1 --------------------------------------------------      0

human             551 cccggcggcggcggcggcggcggcggcgcagggacaagggatggggatct    600

fugu                1 --------------------------------------------------      0

human             601 ggcctcttccttgctttcccgccctcagtacccgagctgtctccttcccg    650

fugu                1 --------------------------------------------------      0

human             651 gggacccgctgggagcgctgccgctgcgggctcgagaaaagggagcctcg    700

fugu                1 --------------------------------------------------      0

human             701 ggtactgagaggcctcgcctggggggaaggccggagggtgggcggcgcgcg    750

fugu                1 --------------------------------------------------      0

human             751 gcttctgcggaccaagtcggggttcgctaggaacccgagacggtccctgc    800

fugu                1 --------------------------------------------------      0

human             801 cggcgaggagatcatgcgggatgagatgggggtgtggagacgcctgcaca    850

fugu                1 --------------------------------------------------      0

human             851 atttcagcccaagcttctagagagtggtgatgacttgcatatgagggcag    900

fugu                1 --------------------------------------------------      0

human             901 caatgcaagtcggtgtgctccccattctgtgggacatgacctggttgctt    950

fugu                1 --------------------------------------------------      0

human             951 cacagctccgagatgacacagacttgcttaaaggaagtgactattgtgac   1000

fugu                1 --------------------------------------------------      0

human            1001 ttgggcatcacttgactgatggtaatcagttgtctaaagaagtgcacaga   1050

fugu                1 --------------------------------------------------      0

human            1051 ttacatgtccgtgtgctcattgggtctatctggccgcgttgaacaccacc   1100

fugu                1 --------------------------------------------------      0

human            1101 aggctttgtattcagaaacaggagggaggtcctgcactttcccaggaggg   1150

fugu                1 --------------------------------------------------      0

human            1151 gtggccctttcagatgcaatcgagattgttaggctctgggagagtagttg   1200

fugu                1 --------------------------------------------------      0

human            1201 cctggttgtggcagttggtaaatttctattcaaacagttgccatgcacca   1250

fugu                1 --------------------------------------------------      0

human            1251 gttgttcacaacaagggtacgtaatctgtctggcattacttctacttttg   1300

fugu                1 --------------------------------------------------      0

human            1301 tacaaaggatcaaaaaaaaaaaagatactgttaagatatgatttttctca   1350

fugu                1 --------------------------------------------------      0

human            1351 gactttgggaaacttttaacataatctgtgaatatcacagaaacaagact   1400

fugu                1 --------------------------------------------------      0
```

```
fugu              1 --------------------------------------------------      0

human          1401 atcatataggggatattaataacctggagtcagaatacttgaaatacggt   1450

fugu              1 --------------------------------------------------      0

human          1451 gtcatttgacacgggcattgttgtcaccacctctgccaaggcctgccact   1500

fugu              1 --------------------------------------------------      0

human          1501 ttaggaaaaccctgaatcagttggaaactgctacatgctgatagtacatc   1550

fugu              1 --------------------------------------------------      0

human          1551 tgaaacaagaacgagagtaattaccacattccagattgttcactaagcca   1600

fugu              1 --------------------------------------------------      0

human          1601 gcatttacctgctccaggaaaaaattacaagcaccttatgaagttgataa   1650

fugu              1 --------------------------------------------------      0

human          1651 aatattttgtttggctatgttggcactccacaatttgctttcagagaaac   1700

fugu              1 --------------------------------------------------      0

human          1701 aaagtaaaccaaggaggacttctgtttttcaagtctgccctcgggttcta   1750

fugu              1 --------------------------------------------------      0

human          1751 ttctacgttaattagatagttcccaggaggactaggttagcctacctatt   1800

fugu              1 --------------------------------------------------      0

human          1801 gtctgagaaacttggaactgtgagaaatggccagatagtgatatgaactt   1850
                                                                |.||
fugu              1 --------------------------------------------gtac--      4

human          1851 caccttccagtcttccctgatgttgaagattgagaaagtgttgtgaactt   1900
                      ||||           |||| ||.|.||             ||.|.|||
fugu              5 --cctt-----------tgat-ttcatga-------------gttagctt     27

human          1901 tctggtactgtaaacagttca----ctgtccttgaagtggtcctgggcag   1946
                    |.|.|           |||||    ..|.|.|.|||||       .|||
fugu             28 ttttg-----------gttcaggcggggacatagaagt-------cgca-     58

human          1947 ctcctgttgtggaaa-gtggacggtttaggatcctgc--ttctctttggg   1993
                             ||.||| ||    |||||  ||.|.|.|  |||||.||
fugu             59 ---------tgcaaacgt----ggttt--gagctttcatttctcctt---     90

human          1994 ctgggagaaaataaacagcatggttacaagtattgagagccaggttggag   2043
                              |||
fugu             91 ----------ata-------------------------------------     93

human          2044 aaggtggcttacacctgt----aatgccagagctttgggaggcggaggca   2089
                         |.||.||.|||.|    |||||..||   ||||           |
fugu             94 -----gactgaccccttttttcaatgcggga---ttgg-----------a    124

human          2090 agaggatcacttgaagccaggagttcaagctcaacctgggcaacgtagac   2139
                    ||||.|            ||||   |||
fugu            125 agagta------------agga---caa----------------------    137

human          2140 cctgtctctacaaaaaattaaaaacttagccgggcgtggtgatgtgcacc   2189
                                      |||.||||        ||| ||||.||
fugu            138 ------------------taataact--------cgt-gtgaggt-----    155

human          2190 tgtagtcct-agctacttgggaggctgaggcaggagggtcatttgagc--   2236
                        .|||| ||             |||.| ||||||| |||.|||.|
fugu            156 ----ctcctcag-------------tgaag-aggaggg-catgtgatcat    186

human          2237 --ccaagagtttgaagttaccgagagctatgatcctgccagtgcattcca   2284
                      |||||||                   |||        |.|||.|
fugu            187 agccaagag-------------------atg--------aatgcgt----    205

human          2285 gcctggatgacaaaacgagaccctgtctctaaaaaacaagaagtgaggc   2334
                       |||               |||.|                      ||
```

```
                         |||               |||.|                      ||
fugu              206 ---tgg---------------ccttt----------------------gc    215

human            2335 tttatgattgtagaattttcactacaatagcagtggaccaaccacctttc   2384
                      |.|||     |||                |.|||||          ||.|
fugu              216 tgtat-----tag----------------gaagtgg----------ttgc    234

human            2385 taaataccaatcagggaagagatggttgattttttaacagacgtttaaag   2434
                                    .||.||||||||.|  ||.|.|.|||..||||.|.|
fugu              235 --------------agatgagatggtgg--ttgtcatcagtggtttgatg    268

human            2435 aaaaagcaaaacctcaaacttagcactctactaacagtttt-agcagatg   2483
                             |.|.|.||||||           ||..||.|||| |.||
fugu              269 -------acatcatcaaac-----------ctgccatttttcaaca----    296

human            2484 ttaattaatgtaatcatgtctgcatgtatgggattatttccagaaagtgt   2533
                       |.|||       |||||.||
fugu              297 -tcatt-------tcatggct-----------------------------    309

human            2534 attgggaaacctctcatgaaccctgtgagcaagccaccgtctcactcaat   2583
                       ||||      |.||.||.||.||||  .||.|||||            |
fugu              310 -ttgg------tgtcctggacactgt--tcaggccac------------t    338

human            2584 ttgaatcttggcttccctcaaaagactggctaatgtttggtaactctctg   2633
                      |    .|||  ||||||        |||.|     ||||.|     ||||
fugu              339 t----cctt--cttccc--------ctgac-----tttgct-----tctg    364

human            2634 ---gagtagacagcactacatgtacgtaagataggtacat----------   2670
                         |.||| |.||||  |||       ||.|||.||.|||
fugu              365 aaagtgta-aaagca--aca-------aaaatatgtccatctgcaatgaa    404

human            2671 aaacaactattggttttgagctgatttttt------tcagctgcatttgc   2714
                      ||.|||      ||..||||||  ||.|||      ||.|| ||...||
fugu              405 aagcaa------gtgatgagct--ttgtttctgagatcggc--caagagc    444

human            2715 atgtatggatttttctcaccaaag-acgat--gacttcaagtattagtaa   2761
                      |   .|||  |||||   |.|||| .|.||  |||  |||..|.|||...
fugu              445 a---ctgg--ttttc---ctaaagttctatgggac--caacaagtagcgg    484

human            2762 aataattgtacagc-tctcctgattat-acttctctgt------gacatt   2803
                      |||..|      || |||.||  |||| ||||.|.|||      ||.| .
fugu              485 aatctt------gcttcttct--ttataacttatatgtcctaaagata-c    525

human            2804 tcatttcccaggctatttcttttggtaggatttaaaact-----aagcaa   2848
                      ||||.|||             |||..||      |||||     ||.|||
fugu              526 tcatatcc-------------ttgtcag------aaactgcttgaatcaa    556

human            2849 ttcagtatgatctttgtcct-tcattttctttcttattctttttgtttgt   2897
                      ||          .||.|||| .||||||..
fugu              557 tt----------gttttcctcacattttag--------------------    576

human            2898 ttgtttgtttgtttttttcttgaggcagagtctctctctgtcgcccaggc   2947

fugu              577 --------------------------------------------------    576

human            2948 tggagtgcagtggcgccatctcagctcattgcaacctctgccacctccgg   2997

fugu              577 --------------------------------------------------    576

human            2998 gttcaagagattctcctgcctcagcctcccgagtagctgggattacaggt   3047

fugu              577 --------------------------------------------------    576

human            3048 gtccaccaccacacccggctaattttttgtatttttagtagaggtggggt   3097

fugu              577 --------------------------------------------------    576

human            3098 ttcaccatgttggccaggctggtcttgagctcctgacctcaggtgatcca   3147

fugu              577 --------------------------------------------------    576

human            3148 cctgcctcggcctaccaaagagctgggataacaggtgtgacccaccatgc   3197

fugu              577 --------------------------------------------------    576

human            3198 ccggcccattttttttttcttattctgttaggagtgagagtgtaactagc   3247
```

```
fugu              577 --------------------------------------------------   576

human            3248 agtataatagttcaattttcacaacgtggtaaaagtttccctataattca   3297

fugu              577 --------------------------------------------------   576

human            3298 atcagattttgctccagggttcagttctgttttaggaaatacttttattt   3347

fugu              577 --------------------------------------------------   576

human            3348 tcagtttaatgatgaaatattagagttgtaatattgcctttatgattatc   3397

fugu              577 --------------------------------------------------   576

human            3398 caccttttttaacctaaaagaatgaaagaaaaatatgtttgcaatataatt   3447

fugu              577 --------------------------------------------------   576

human            3448 ttatggttgtatgttaacttaattcattatgttggcctccagtttgctgt   3497

fugu              577 --------------------------------------------------   576

human            3498 tgttagttatgacagcagtagtgtcattaccatttcaattcagattacat   3547

fugu              577 --------------------------------------------------   576

human            3548 tcctatatttgatcattgtaaactgactgcttacattgtattaaaaacag   3597

fugu              577 --------------------------------------------------   576

human            3598 tggatattttaaagaagctgtacggcttatatctagtgctgtctcttaag   3647

fugu              577 --------------------------------------------------   576

human            3648 actattaaattgatacaacatatttaaaagtaaatattacctaaatgaat   3697

fugu              577 --------------------------------------------------   576

human            3698 ttttgaaattacaaatacacgtgttaaaactgtcgttgtgttcaaccatt   3747

fugu              577 --------------------------------------------------   576

human            3748 tctgtacatacttagagttaactgttttgccaggctctgtatgcctactc   3797

fugu              577 --------------------------------------------------   576

human            3798 ataatatgataaaagcactcatctaatgctctgtaaatagaagtcagtgc   3847

fugu              577 --------------------------------------------------   576

human            3848 tttccatcagactgaactctcttgacaagatgtggatgaaattctttaag   3897

fugu              577 --------------------------------------------------   576

human            3898 taaaattgtttactttgtcatacatttacagatcaaatgttagctcccaa   3947

fugu              577 --------------------------------------------------   576

human            3948 agcaatcatatggcaaagataggtatatcatagtttgcctattagctgct   3997

fugu              577 --------------------------------------------------   576

human            3998 ttgtattgctattattataaatagacttcacagttttagacttgcttagg   4047

fugu              577 --------------------------------------------------   576

human            4048 tgaaattgcaattctttttactttcagtcttagataacaagtcttcaatt   4097

fugu              577 --------------------------------------------------   576

human            4098 atagtacaatcacacattgcttaggaatgcatcattaggcgattttgtca   4147

fugu              577 --------------------------------------------------   576

[illegible]
```

```
human           4148 ttatgcaaacatcatagagtgtacttacacaaacctagatagtatagcct   4197

fugu             577 --------------------------------------------------    576

human           4198 ttatgtacctaggccgtatggtatagtctgttgctcctaggccacaaacc   4247

fugu             577 --------------------------------------------------    576

human           4248 tgtacaactgttactgtactgaatactatagacagttgtaacacagtggt   4297

fugu             577 --------------------------------------------------    576

human           4298 aaatatttatctaaatatatgcaaacagagaaaaggtacagtaaaagtat   4347

fugu             577 --------------------------------------------------    576

human           4348 ggtataaaagataatggtatacctgtgtaggccacttaccacgaatggag   4397

fugu             577 --------------------------------------------------    576

human           4398 cttgcaggactagaagttgctctgggtgagtcagtgagtgagtggtgaat   4447

fugu             577 --------------------------------------------------    576

human           4448 taatgtgaaggcctagaacactgtacaccactgtagactataaacacagt   4497

fugu             577 --------------------------------------------------    576

human           4498 acgctgaagctacaccaaatttatcttaacagtttttcttcaataaaaaa   4547

fugu             577 --------------------------------------------------    576

human           4548 ttataactttttaactttgtaaactttttaatttttttaacttttaaaata   4597

fugu             577 --------------------------------------------------    576

human           4598 cttagcttgaaacacaaatacattgtatagctatacaaaaatatttttttc   4647

fugu             577 --------------------------------------------------    576

human           4648 tttgtatccttattctagaagctttttttctattttctattttaaattttt   4697

fugu             577 --------------------------------------------------    576

human           4698 tttttacttgttagtcgtttttgttaaaaactaaaacacacacactttc   4747

fugu             577 --------------------------------------------------    576

human           4748 acctaggcatagacaggattaggatcatcagtatcactcccttccacctc   4797

fugu             577 --------------------------------------------------    576

human           4798 actgccttccacctccacatcttgtcccactggaaggtttttaggggcaa   4847

fugu             577 --------------------------------------------------    576

human           4848 taacacacatgtagctgtcacctatgataacagtgctttctgttgaatac   4897

fugu             577 --------------------------------------------------    576

human           4898 ctcctgaaggacttgcctgaggctgttttacatttaacttaaaaaaaaaa   4947

fugu             577 --------------------------------------------------    576

human           4948 aaagtagaaggagtgcactctaaaataacaataaaaggcatagtatagtg   4997

fugu             577 --------------------------------------------------    576

human           4998 aatacataaaccagcaatgtagtagtttattatcaagtgttgtacactgt   5047

fugu             577 --------------------------------------------------    576

human           5048 aataattgtatgtgctatactttaaataacttgcaaaatagtactaagac   5097

fugu             577 --------------------------------------------------    576
```

```
human           5098 cttatgatggttacagtgtcactaaggcaatagcatattttcaggtccat   5147

fugu             577 --------------------------------------------------    576

human           5148 tgtaatctaatgggactaccatcatatatgcagtctaccattgactgaaa   5197

fugu             577 --------------------------------------------------    576

human           5198 cgttacatggcacataactgtatttgcaagaatgatttgttttacattaa   5247

fugu             577 --------------------------------------------------    576

human           5248 tatcacataggatgtacctttttagagtggtatgtttatgtggattaaga   5297

fugu             577 --------------------------------------------------    576

human           5298 tgtacaagttgagcaaggggaccaagagccctgggttctgtcttggatgt   5347

fugu             577 --------------------------------------------------    576

human           5348 gagcgtttatgttcttctcctcatgtctgttttctcattaaattcaaagg   5397

fugu             577 --------------------------------------------------    576

human           5398 cttgaacgggccctatttagcccttctgttttctacgtgttctaaataac   5447

fugu             577 --------------------------------------------------    576

human           5448 taaagcttttaaattctagccatttagtgtagaactctctttgcagtgat   5497

fugu             577 --------------------------------------------------    576

human           5498 gaaatgctgtattggtttcttggctagcatattaaatatttttatctttg   5547

fugu             577 --------------------------------------------------    576

human           5548 tcttgatacttcaatgtcgttttaaacatcaggatcgggcttcagtattc   5597

fugu             577 --------------------------------------------------    576

human           5598 tcataaccagagagttcactgaggatacaggactgtttgcccatttttttg   5647

fugu             577 --------------------------------------------------    576

human           5648 ttatggctccagacttgtggtatttccatgtcttttttttttttttttt   5697

fugu             577 --------------------------------------------------    576

human           5698 tttgaccttttagcggctttaaagtatttctgttgttaggtgttgtatta   5747

fugu             577 --------------------------------------------------    576

human           5748 cttttctaagattacttaacaaagcaccacaaactgagtggctttaaaca   5797

fugu             577 --------------------------------------------------    576

human           5798 acagcaatttattctctcacaattctagaagctagaagtccgaaatcaaa   5847

fugu             577 --------------------------------------------------    576

human           5848 gtgttgacaggggcatgatcttcaagagagaagactctttccttgcctct   5897

fugu             577 --------------------------------------------------    576

human           5898 tcctggcttctggtggttaccagcaatcctgagtgttcctttcttgcctt   5947

fugu             577 --------------------------------------------------    576

human           5948 gtagtttcaacaatccagtatctgccttttgtcttcacatggctgtctac   5997

fugu             577 --------------------------------------------------    576

human           5998 catttgtctctgtgtctccaaatctctctccttataaacacagcagttat   6047

fugu             577 --------------------------------------------------    576
```

```
human             6048 tggattaggccccactctaatccagtatgaccccattttaacatgattac   6097

fugu               577 --------------------------------------------------    576

human             6098 acttatttctagataaggtcacattcacgtacaccaagggttaggaattg   6147

fugu               577 --------------------------------------------------    576

human             6148 aacatatctttttgggggacacaattcaacccacaagtgtcagtctctag   6197

fugu               577 --------------------------------------------------    576

human             6198 ctgagcctttcccttcctgtttttctcctttttagttgctatgggttagg   6247

fugu               577 --------------------------------------------------    576

human             6248 ggccaaatctccagtcatactagaattgcacatggactggatatttggga   6297

fugu               577 --------------------------------------------------    576

human             6298 atactgcgggtctattctatgagctttagtatgtaacatttaatatcagt   6347

fugu               577 --------------------------------------------------    576

human             6348 gtaaagaagccctttttaagttatttctttgaatttctaaatgtatgcc   6397

fugu               577 --------------------------------------------------    576

human             6398 ctgaatataagtaacaagttaccatgtcttgtaaaatgatcatatcaaca   6447

fugu               577 --------------------------------------------------    576

human             6448 aacatttaatgtgcacctactgtgctagttgaatgtctttatcctgatag   6497

fugu               577 --------------------------------------------------    576

human             6498 gagataacaggattccacatctttgacttaagaggacaaaccaaatatgt   6547

fugu               577 --------------------------------------------------    576

human             6548 ctaaatcatttggggttttgatggatatctttaaattgctgaacctaatc   6597

fugu               577 --------------------------------------------------    576

human             6598 attggtttcatatgtcattgtttag   6622

fugu               577 -------------------------    576


#---------------------------------------
#---------------------------------------
</pre></body></html>
```
