# Supplementary material for: A comparative bioinformatic analysis of C9orf72
Source: PeerJ. 2018 Feb 19;6:e4391. doi: 10.7717/peerj.4391 (PMC5822839; doi:10.7717/peerj.4391)
Supplement: Figure S6 — Sequence alignment of Intron 1 sequences using EBI’s EMBOSS Needle (Rice, Longden & Bleasby, 2000). [file peerj-06-4391-s008.pdf]

```
<html><head></head><body><pre style="word-wrap: break-word; white-space: pre-
wrap;">########################################
# Program: needle
# Rundate: Wed 24 Jan 2018 12:56:22
# Commandline: needle
#    -auto
#    -stdout
#    -asequence emboss_needle-I20180124-125619-0400-7777544-p2m.asequence
#    -bsequence emboss_needle-I20180124-125619-0400-7777544-p2m.bsequence
#    -datafile EDNAFULL
#    -gapopen 25.0
#    -gapextend 0.5
#    -endopen 10.0
#    -endextend 0.5
#    -aformat3 pair
#    -snucleotide1
#    -snucleotide2
# Align_format: pair
# Report_file: stdout
########################################

#=======================================
#
# Aligned_sequences: 2
# 1: human
# 2: mouse
# Matrix: EDNAFULL
# Gap_penalty: 25.0
# Extend_penalty: 0.5
#
# Length: 10983
# Identity:    2870/10983 (26.1%)
# Similarity:  2870/10983 (26.1%)
# Gaps:        6829/10983 (62.2%)
# Score: 2781.5
#
#
#=======================================

human              1 gtgtgtcagccgtccctgctgcccggttgcttctcttttgggggcggggt     50

mouse              1 --------------------------------------------------      0

human             51 ctagcaagagcaggtgtgggtttaggaggtgtgtgtttttgtttttccca    100

mouse              1 --------------------------------------------------      0

human            101 ccctctctccccactacttgctctcacagtactcgctgagggtgaacaag    150

mouse              1 --------------------------------------------------      0

human            151 aaaagacctgataaagattaaccagaagaaaacaaggagggaaacaaccg    200

mouse              1 --------------------------------------------------      0

human            201 cagcctgtagcaagctctggaactcaggagtcgcgcgctaggggccgggg    250

mouse              1 --------------------------------------------------      0

human            251 ccggggccggggcgtggtcggggcgggcccgggggcgggcccggggcggg    300

mouse              1 --------------------------------------------------      0

human            301 gctgcggttgcggtgcctgcgcccgcggcggcggaggcgcaggcggtggc    350

mouse              1 --------------------------------------------------      0

human            351 gagtgggtgagtgaggaggcggcatcctggcgggtggctgtttggggttc    400
                           |||||||||..|...|......||.|||..||||....||  ||
mouse              1 ------gtgagtgagacgcgcgggcggaggggggctgctgccacgg--tc     42

human            401 ggctgccgggaag---aggcgcgggtagaagcgggg----gctctcctca    443
                     ||||..||||..|   .|...||||||..|||||||    ..||||||..
mouse             43 ggctcgcgggccggccggctccgggtaccagcggggtttttttctccttc     92
```

```
human       444 gagctcgacgcattttactttccctctcatttctctgaccgaagctggg   493
                |||.|..||             .||||.|..|.|.|.|..||||
mouse        93 gaggtgaac-------------tcctccctgtcccccgggcgaa------   123

human       494 tgtcgggctttcgcctctagcgactggtggaattgcctgcatccgggccc   543
                                                           .|.||||
mouse       124 -------------------------------------------agagccc   130

human       544 cgggcttcccggcggcggcggcggcggcggcgcagggacaagggatg   593
                ..|||.|..|.|..|..||||.|||.|||||||.||.|    |.||||||
mouse       131 ttggccttgcaggagttgcgggggccgcggcggtgcgg----aggggatg   176

human       594 gggatctggcctcttccttgctttcc-----cgccctcagtacccgagct   638
                ||||| .||||||.||.|||||.|||     |||.|.|.|..|||||.|.
mouse       177 gggat-gggcctcatctttgctgtccgcccgcgctccccgatcccgaccc   225

human       639 gtctccttccc-----------ggggacccgctgggagcgctgccgctgc   677
                |...|.|..||           |||.||||.|.|||||..|.||....||
mouse       226 ggagcgtctcccgggcccttgagggaaccctccgggagtacggcgagcgc   275

human       678 gggctcgagaaaagggagcctcgggtactgagaggcctcgcctgggggaa   727
                ||.|.|.|........|||||  ||..|..||.|||||.|||.||
mouse       276 ggcccccaccgccacaagcct--gggccccaggggcctggcccgg-----   318

human       728 ggccggagggtgggcggcgcgcggcttctgcggaccaagtcggggttcgc   777
                           .|.|.||..|.||.|.|||| ||||.||||.||..||.|
mouse       319 -----------cgacagctggtgggtcctgc-gacccagtcaggtctccc   356

human       778 taggaacccgagacggtccctgccggcgaggagatcatgcgggatgagat   827
                           ||.||||||.|||.|.|||||||         |.|.|..
mouse       357 -----------gagggtccccgcccgggaggaga---------aagcgcc   386

human       828 gggggtgtggag-------------------acgcctgcacaatttcagc   858
                ||.||..|||||                   ||||..|||..||||| ||
mouse       387 ggtgggatggagtaaggacggacagaacaacacgcaggcaggatttc-gc   435

human       859 ccaagcttctagagagtggtgatg---acttgcatatgagggcagcaatg   905
                ..|||.||..|..|||||..||||   ||||.|||    ||||.||.|..
mouse       436 agaagtttgcaaggagtgcggatgcccacttacat----gggctgctact   481

human       906 caagtcggtgtgctcccca-ttctgtgggacatgacctggttgcttcaca   954
                |....|.|..||.|||||| |||||||||||.||||||||||||.|||||
mouse       482 cttaccaggttgttccccagttctgtgggacgtgacctggttgcctcaca   531

human       955 gctccgagatgacacagacttgcttaaaggaagtgactattgtgacttgg  1004
                ||||||.|.|...|||||||| .|||||||||||||.|||||||||||||
mouse       532 gctccgcggttgtacagactt-attaaaggaagtgaccattgtgacttgg   580

human      1005 gcatcacttgactgatggtaatcagttg--tctaaagaagtgcacagatt  1052
                ||||||||||||||||||||||||||||  ...|.||||||||||.||||
mouse       581 gcatcacttgactgatggtaatcagttgcagagagagaagtgcactgatt   630

human      1053 acatgtccgtgtgctcattgggtctatctggccgcgttgaacaccaccag  1102
                |..|    .|||.|.||..||||||.||||||
mouse       631 aagt----ctgtccacacagggtctgtctggc------------------   658

human      1103 gctttgtattcagaaacaggagggaggtcctgcactttcccaggagg---  1149
                                ||||||        ||||.||.||..|||||
mouse       659 ----------------caggag--------tgcatttgcctgggagggat   684

human      1150 -ggtggccctttcagatgcaatcgagattgttaggctctgggagagtagt  1198
                 |||.||.|||||.|.||   |.|.||.|.|||||||||.|.|||||
mouse       685 tggttgcgctttctggtg---tggggactattaggctcttgtagagt---   728

human      1199 tgcctggttgtggcagttgg-taaatttctattcaaacagttgccatgca  1247
                   .|.||...|||||.||| |||||||||..|.|.||.|||.||.|.|.
mouse       729 ---tttgtcccggcagatggataaatttcttgttacactgttcccgttcg   775

human      1248 ccagttgttcacaacaa-gggtacgtaatctgtct-ggcattacttctac  1295
                .||...|||.|.||.|| ||||||..|.||||||| .|.|.|||||.|||
mouse       776 tcaccagttgagaaaaacgggtacacagtctgtctcagtagtacttttac   825

human      1296 ttttgtacaaaggatcaaaa--aaaaaaaagatactgttaagatatgatt  1343
                |||.....||.||..|||||  .|....||.||||| ||||||||..||.
mouse       826 tttatattaaggggggaaaagggagtggaaaatagt-ttaagatagaatg   874
```

```
mouse         826 tttatattaagggcccaaaagggactggaaaatact-ttaagatagaatc   874

human        1344 tttctcagactttgggaaacttttaacataatctgtga------------   1381
                  .||.....|| ||||.||||||     |.|||.||.||
mouse         875 gttagtccac-ttggaaaactt-----aaaatatgagagagagagggggg   918

human        1382 ------------------------atatcacagaaacaagactatcatat   1407
                                          |.|..|..|||..||||.......|.
mouse         919 ggggagagagagagagagagagagagagaaaggaaggaagaaggaggaag   968

human        1408 agggg-------------------atattaataacctggagtcagaatac   1438
                  |||.|                   ||.|||||||..||||.||||||||.
mouse         969 aggaggaggaaagagattgagattatgttaataatatggaatcagaatat   1018

human        1439 ttgaaatacggt---------gtcatttgacacgggcat-----------   1468
                  ||||||||..||         .|||.||.|...||.|||
mouse        1019 ttgaaatatagtaagcgtcccctcagttaaagaggacattccaggaggcc   1068

human        1469 --------------------------------------------------   1468

mouse        1069 cccagtatagcctgaaatctcaggaaacgcctacatacacccatcgtgtg   1118

human        1469 --------tgttgtcac---------------------------------   1477
                          ||||.||.|
mouse        1119 gatataggtgttttcccttcattacatttcatacacagatgttaaagttt   1168

human        1478 --------------------------------------------------   1477

mouse        1169 agaaagtaggcacaataagagattacaaataactgataataaagtcgagc   1218

human        1478 --------------------------------------------------   1477

mouse        1219 cattgcagctgctctgtaaaagtcctgtgaatgtgatcgctttgtgtttc   1268

human        1478 -------------------cacctctgccaag------------------   1490
                                     ||||.|||..|||
mouse        1269 aaagtaacttactgtacttcacccctgttaagcaaaacaagattcacctg   1318

human        1491 --------------------------------------------------   1490

mouse        1319 aacgcaggcaccttggtaccttggcagacaccagatctgataaccaagag   1368

human        1491 --------------------------------------------------   1490

mouse        1369 gatggagaagtagtggcagacagtgtggagagcatgaatatgctagacaa   1418

human        1491 --------------------------------------------------   1490

mouse        1419 aagggtgaatcataacctaggagcagaaagcaggtatttcatcatcctcc   1468

human        1491 --------------------------------------------------   1490

mouse        1469 acagtaaaaacctatgtcacgtaaaaaacctacaagtagtttttctttta   1518

human        1491 ----------------gcctgc----------------------------   1496
                                  ||.|||
mouse        1519 ctctttttgaatgaaagcttgctacaggcactgaaagttaaaataatctg   1568

human        1497 --------------------------------------------------   1496

mouse        1569 tggatcaggaggaacaggggttttctgtctgagtcactgctgactagcac   1618

human        1497 ---------------cactttaggaaaccctgaatcagttggaaactgc   1531
                                 ||||.|.|||||.|||.||.|||||||||||||.|
mouse        1619 ctcagtgaccattggcactgtgggaaaccccagagtcagttggaaacttc   1668

human        1532 taca-------------tgctgatagtacatctgaaac-aagaacgagag   1567
                  .|.|             ||.|..||.|.|||...|.|| ||.||..||||
mouse        1669 gaaactaaaggtgacggtgttcttatttcatagaacacaaaaaataagag   1718

human        1568 taattaccacat----tccagattgttcactaagccagcattt-acctgc   1612
                  ...||||..|.|    |.||||.||..||.|.|.|.||||||| |..|.|
mouse        1719 gggttacagcctgcgctgcagactggacattcaacaagcatttaaatttc   1768

human        1613 tccaggaaaaaattacaagcaccttatgaagttgataaaatattttgttt   1662
                  |....||.|||..||.|...|.|||...||||||.|||||||.|.|||||
```

```
mouse         1769 tgggagacaaatgtaaatataactttaaaagttggtaaaatactctgttt   1818

human         1663 ggctatgttggcactccacaatttgctttcagagaaacaaag------ta   1706
                   ||||||||||||..||||...||||||||.|||.||..|..|      ||
mouse         1819 ggctatgttggccatccaatgtttgcttttagaaaatgactgaatggata   1868

human         1707 aaccaaggaggacttctgtttttcaagtctgccctcgggttctattctac   1756
                   ||         ||.|||.|.|||..||.|||||||.|....|.||
mouse         1869 aa---------acgtctatcttttgagcctgccctagacccccat-----   1904

human         1757 gttaattagatagttcccaggaggactaggttagcctacctattgtctga   1806
                   |||.|.|..|||.|..|||  ||...||||||||||       .|.||||
mouse         1905 gttgagtgaatactgtcca--agtgttaggttagcc-------ggcctga   1945

human         1807 gaaacttggaactgtgagaaatggccagatagtgatatga--acttcacc   1854
                   ||||||||||.||..|..|.|||||....|..||.|.|.|  |.|...|.
mouse         1946 gaaacttggatctaggcaagatggcacagtcctggtgtcatgagtatgca   1995

human         1855 ttccagtcttccctgatgttga----------agattgagaaagtgttgt   1894
                   |...|||.||..||||..||||          |||.|||.||||
mouse         1996 tgtgagttttggctgaaattgaacatttgtagagaatgacaaag------   2039

human         1895 gaactttctggtactgtaaacagttcactgtccttgaagtggtcctgggc   1944
                         .|||||...|.||..|||.|||||||.||..|||||||.|||..
mouse         2040 ------gctggtctggcaagtagtccactgtctttacagtggtcttggtt   2083

human         1945 agctcctgttgtggaaagtggac-ggtttagg---atcctgcttctcttt   1990
                   ||.|||||||..|...||.||.| ||||.|.|   .||||||..|||||
mouse         2084 agttcctgtttggctgagagggctggttgatggctgtcctgcccctctt-   2132

human         1991 gggctgggagaaaataaacagcatggttacaagtattgagagccaggttg   2040

mouse         2133 --------------------------------------------------   2132

human         2041 gagaaggtggcttacacctgtaatgccagagctttgggaggcggaggcaa   2090

mouse         2133 --------------------------------------------------   2132

human         2091 gaggatcacttgaagccaggagttcaagctcaacctgggcaacgtagacc   2140

mouse         2133 --------------------------------------------------   2132

human         2141 ctgtctctacaaaaaattaaaaacttagccgggcgtggtgatgtgcacct   2190

mouse         2133 --------------------------------------------------   2132

human         2191 gtagtcctagctacttgggaggctgaggcaggagggtcatttgagcccaa   2240

mouse         2133 --------------------------------------------------   2132

human         2241 gagtttgaagttaccgagagctatgatcctgccagtgcattccagcctgg   2290

mouse         2133 --------------------------------------------------   2132

human         2291 atgacaaaacgagaccctgtctctaaaaaacaagaagtgagggctttatg   2340
                                                .|.|.|||||...||.|||||
mouse         2133 -----------------------------cccacaagtggaagccttatg   2153

human         2341 attgtagaatt-ttcactacaatagcagtggaccaaccacctttctaaat   2389
                      |||.|||| ||.|..|||.|||||||.|.|.||..|.|||.||.||
mouse         2154 ---gtataattcttgatcacagtagcagtaggcaaatgaacttcctcaa-   2199

human         2390 accaatcagggaag----------------------agatggttgatttt   2417
                   |.||..|.||.|||                      ...|..||..||||
mouse         2200 agcagcctggaaagctgatttttttttctttctttctctttttttttttt   2249

human         2418 ttaacagacgtttaaagaaaaagcaaa--acctcaaacttagcactctac   2465
                   ||..||.|.|.|||||||||||.||||  .|.|||||..|..||.|||.|
mouse         2250 ttttcacaaggttaaagaaaaaacaaagggcttcaaatgtgccagtctgc   2299

human         2466 taacagttttagcagatgttaattaatgtaatcatgtctgcatgtatggg   2515
                   |||||||.|||  |.|||||.|||||..|||          ||..|....
mouse         2300 taacagtgtta--acatgtttattaacataa----------ataaacttt   2337

human         2516 attatttccagaaagtgtattgggaaacctctcatgaaccctgtgagc--   2563
```

```
              ||||.||...|.||  |||||||..||.|.|||.|||.|||||....|
mouse    2338 attagtttttggaa--gtattggttaagccctcgtgacccctgaactcgg  2385

human    2564 -------------aagccaccgtctcactc-aatttgaatcttggcttcc  2599
                           .||.|...|.||||||| ..||||.|...|||||||.
mouse    2386 tttatagagtgatgagtcgtagcctcactctggtttggactctggcttct  2435

human    2600 ctcaaaagac----tggctaatgtttggtaactctctggagtagacagca  2645
              ||||.|||||    ||||||||||    ||||..||||.|||||.||| |
mouse    2436 ctcagaagactctgtggctaatgt----taaccttctgaagtagccag-a  2480

human    2646 ctacatgtacgtaagataggtacataaacaactat-tggttttgagctga  2694
              ..||||.||.|.||               ||.|.| ||...||||..|||
mouse    2481 aaacatataagcaa---------------aagtctgtgaggttgaaatga  2515

human    2695 tttttttcagctgcatttgcatgtatggatttttctcaccaaagacgatg  2744
              .|||||| .||..||||||.||.|..|     |||.|||||   |.|.|.
mouse    2516 atttttt-ggccacatttgtatatggg-----ttcccacca---atgcta  2556

human    2745 acttcaagtattagtaaaataattgt-acagctctcctgattatacttct  2793
              ||||||.||.|||||||.||.|...| ||||||..||||||||.|||||.
mouse    2557 acttcaggtgttagtaatatcagactcacagcttccctgattacacttcg  2606

human    2794 ctgtgacatttcatttcccagg--------ctatttcttttggtaggatt  2835
              ||.|.|.|.||.||||...|||        .|.|..|.|||...|.||||
mouse    2607 ctataagactttattttttaggtcataggaatttcccctttttcatgatt  2656

human    2836 ------------------------taaaactaagcaatt-----------  2850
                                      |||||.||.|..|||
mouse    2657 cctaaatcatgaaataacatagtctaaaaatacggtattcctgaaataaa  2706

human    2851 -----------------------------------cagtatgat------  2859
                                                 ||||.||||
mouse    2707 caatttctaagttttaagctgcgtgctattctgaacagtctgatgccctc  2756

human    2860 -----------------------------------------ctttgtc--  2866
                                                       |||||||
mouse    2757 ttgtagcttttactgtgtcctaccccgggcatggttgattcctttgtcca  2806

human    2867 --------------------------------------------------  2866

mouse    2807 aacatctgtctgttgtatccacactggattgcaccacctgcgtgctagtc  2856

human    2867 ------------------------------cttcattttctttcttattc  2886
                                            |||...|||..|.||||||.
mouse    2857 agtcactcagacattttagttataaggtagcttatatttactccttattt  2906

human    2887 ttttt--------------------------------------------g  2892
              |.|||                                            |
mouse    2907 tatttaataatggcctcatagcaaggcggtaatgatactggtaatttggg  2956

human    2893 tttgtt--------------------------------------------  2898
              ||||.|
mouse    2957 tttgcttaagaggagccatgaagtagttttaaatgaaaaggtgaaaattc  3006

human    2899 --------------------------------------------------  2898

mouse    3007 ccactatagtttggaggggg aggctatactggtactactacgattcacgg  3056

human    2899 --------------------------------------------------  2898

mouse    3057 taagactaaatcttctgtgaaattatgaaggagaaaaagttacactggtc  3106

human    2899 ---------tgtttgtttgtttttttcttga-------------------  2920
                       ||||.|.||..||||.|..|.|
mouse    3107 tggtcttgctgttggattaattttatagttataaccactgtacatgataa  3156

human    2921 --------------------------------ggcagagtct--------  2930
                                              ||||.|.|||
mouse    3157 ataaccctaaaacaatgaatttgtaggtggatggcataatctgaaaacca  3206

human    2931 --------------------------------------------------  2930

mouse    3207 tgttctgagcagttgatggcagcaggctgtgctggaagtgttaggcatat  3256

[illegible]
```

```
human          2931 --------------------------------------------------   2930

mouse          3257 ttatagatttcagcccaagttctgaagaggctggagagatggctcagtgg   3306

human          2931 -----------------------------------ctct-----------   2934
                                                       ||||
mouse          3307 ttaagagtgcttgctattgcagaggacctaggttcctctacaggcaccag   3356

human          2935 --------------------------------------------------   2934

mouse          3357 gcaagcgtgggacacactgagatacatacagacaaaacataaaattaaat   3406

human          2935 --------------------------------------------------   2934

mouse          3407 aaattgtgcataataatactagtaatatatgagtaaaataaggataaata   3456

human          2935 --------------------------------------------------   2934

mouse          3457 cacatcataattaaataaataaattgtaaagttccctagaagtgagggtc   3506

human          2935 --------------------------------------------------   2934

mouse          3507 accaagccattcacaagatggctgcgctgatgcagggatatatgtgaact   3556

human          2935 --------------------------------------------------   2934

mouse          3557 agaaaaaggtcaaacttaacagagaagttccaaggcatgctactgcaggc   3606

human          2935 -----------------------------ctgtcgcc-------------   2942
                                                 |||.||.|
mouse          3607 ttggctagcatgcttgacctgcagaaatgctgacggccactgggaggttt   3656

human          2943 --------------------------------------------------   2942

mouse          3657 tcacaaatgaggaattagaagaactttttttactaatctccagaaaaaaa   3706

human          2943 ---------------------caggctg----------------------   2949
                                         |||.|||
mouse          3707 aaagggaagaagaaactgaagcagcctgtgatgtggaccagaaacgcagt   3756

human          2950 --------------------------------------------------   2949

mouse          3757 gacagtaacatgtgtgacattgcaaaggcatgaaaggacagagctgtgga   3806

human          2950 --------------------------------------------------   2949

mouse          3807 atacagacctcaggtggagctcagcatagagtcattcggggattatgcct   3856

human          2950 --------------------------------------------gagtgc   2955
                                                                ||.|||
mouse          3857 gctgcagcaacaaaaggatgagctcaaaagagacaccgacttctgaatgc   3906

human          2956 agtgg---------------------------------------------   2960
                    |||||
mouse          3907 agtgggtgtttgttttgttttgtttcaaatgaattgggcagaaaactttc   3956

human          2961 ----------------------------------cgccatctcagctcat   2976
                                                      .|.|||||.|||   .
mouse          3957 cagctgtggaagcttctgaaccgtcccttgctgctgacatctaagc---g   4003

human          2977 tgcaacctctgccacctccgggttc------------aagagattctcct   3014
                    |.|....|.|.|||.|||.|.|.||            ||.||||..|||.
mouse          4004 tccgctgtgtcccagctcagtgatctagggtcttccaaacagatggtccg   4053

human          3015 g-------------------------------------------------   3015
                    |
mouse          4054 gtgctgagcactttgaatctcaatcctgagtttctaccacgcctttggcc   4103

human          3016 -------------------------------------------------c   3016
                                                                     |
mouse          4104 atttaattcccagataaaagacacatacaacctttatatttataataaac   4153

human          3017 ctcagcctcccgagtagctgggattacaggtgtcc---------------   3051
                    ||.||.|..|..|..|||||.|...|.|..|||||
mouse          4154 cttagtcagcacaagagctgagcaaatatctgtcctctatgctattatat   4203
```

```
human         3052 --------------------------------------------------   3051

mouse         4204 ctattacccagccaataaccccattctataatttgctgtgcttcatctgg   4253

human         3052 --------------------accaccacacccggctaatttttt---gta   3078
                                       .|||.|.|||..|||.|.|.||||   .|.
mouse         4254 gctgctcttaacttcagtcagccagcccacgtggccattattttaagatt   4303

human         3079 ttttta--------------------------------------------   3084
                   ||||||
mouse         4304 tttttaccccatagtgtcttctcactttactttacatttttctctctctc   4353

human         3085 --------------------------------------------------   3084

mouse         4354 ctcatggttctcctctgaccccaagcctaggaaccctaaaccccacccat   4403

human         3085 ------------------------gtagaggtggggtttcacca------   3104
                                           ||||...|.....|||||||
mouse         4404 gtctcttctgcccatctattggctgtaggcatctttattcaccaatcagg   4453

human         3105 ------tgttggccaggct-------------ggtcttg-----------   3124
                         ||..|||.|||.|             |||||.|
mouse         4454 ataacttggaggcaaggttaagtagtctcctgggtctaggtgctgtctct   4503

human         3125 ---------------------------agctcctgacctc----------   3137
                                              |...||.||||||
mouse         4504 gggagcaaccagtatttagcatagcaaaagaccagacctccacaatgatc   4553

human         3138 -------------------aggtgatccacctgcctcggcctacca----   3164
                                      |.|..|.|.||..||||..|||...||
mouse         4554 actctgaccatcggggcagaaggcacctactagcctgtgccactcacctc   4603

human         3165 -------------------------------------aagagctgggata   3177
                                                        |....|||..||.
mouse         4604 actttgttgaatcacatcttatcctgtagtgtgtatcactgcctgttatc   4653

human         3178 acag------gtgtgaccc-------------------------accatg   3196
                   ||||      |||.|.|||                         ||||||
mouse         4654 acaggaaaaagtgagtcccatcaaataagatgtttcagaaagagaccatg   4703

human         3197 cccggcccattttttttttcttattctgttaggagtga------------   3234
                   ..|.....|||.|..||.|..||..||.|||...||.|
mouse         4704 ttcatataattatcattctggtaagcttttaatggttatattttgttatt   4753

human         3235 --------------------------------------------------   3234

mouse         4754 aatctctttgttcctattttgcaaattataccttacagtaaatatatatg   4803

human         3235 --------------------------------------------------   3234

mouse         4804 catccaatggggtctttgaattcctccccggggagtaggaggactctttg   4853

human         3235 --------------------------------------------------   3234

mouse         4854 aggatgggctgcatttaaagctaaacaacgcaacatgacctttagtcctt   4903

human         3235 -----------------gagtgtaactagcag--------tataatagtt   3259
                                    |||..|||.||..||        ||||||..||
mouse         4904 atagatagcctagagatgagactaaataaaagaaatggtatataatgctt   4953

human         3260 caattttcacaacgtggt--aaaagtttccctataattcaatcagatttt   3307
                   .||.||||.|||...|.|  |||..|||.|||||||.||..|.|||||.|
mouse         4954 taagtttcccaatcagcttaaaagcttttcctataaatctttaagattat   5003

human         3308 gctccagggttcagttctgttttaggaaatacttttattttcagtttaat   3357
                   ||||..|||.|||.|.|||.||.|.|||...|||||.||
mouse         5004 gctctggggctcaatactgcttcaagaagggcttttctt-----------   5042

human         3358 gatgaaatattagagttgtaatattgcctttatgattatccaccttttta   3407
                                          |||..||||..|||||.||||||||||
mouse         5043 -----------------------ttgtatttagaattattcaccttttta   5069

human         3408 acctaaa----agaatgaaagaaaaatatgtttgcaatataattttatgg   3453
                   |.|.|||    |.||||.||.|.||||||||||||||.||||||||||.
mouse         5070 aacaaaaggagaaaatggaatagaaatatgtttgcaacataattttatga   5119
```

```
human     3454 ttgtatgttaacttaattcattatgttggcctccagtttgctgttgttag   3503
               .|.|.||||.|.||......||.|||.|||||.||||||||||.|||||.
mouse     5120 ctatgtgtttatttcgcgtgttctgtgggcctgcagtttgctgctgttaa   5169

human     3504 ttatgacagcagtagtgtcattaccatttcaattcagattacattcctat   3553
               |.|.||||.||||.|...||.|||..||||.|.|||||||||||| ||.|
mouse     5170 tgaggacaacagtggcaccaatacagtttccactcagattacatt-ctct   5218

human     3554 atttgatcattgtaaactgactgcttacattgtattaaaaacagtggata   3603
               .||...|...||.||.|||.|..| |.||.||.....||||.|||...||
mouse     5219 gttccctttctgaaagctgccctc-tccactgggcccaaaagagtcagta   5267

human     3604 ttttaaagaagctgtacggcttatatctagtgctgtctcttaagactatt   3653
               |.|||||.|||||||||..||||.|| .|.....|||||||.||||||.|
mouse     5268 tcttaaacaagctgtacaacttagat-aaccatggtctcttcagactagt   5316

human     3654 aaattgatacaacatatttaaaagtaaatattacctaaatgaatttttga   3703
               .||||||   .|.|||||.|||||||||||.||||.||.|||||||.|||
mouse     5317 taattga---catatattaaaaagtaaatagtaccaaagtgaatttctga   5363

human     3704 aattacaaatacacgtgttaaaactgtcgttgtgttcaaccatttctgta   3753
               |||||.||||..||.|.|.||||||.|     .|.|.|||.||
mouse     5364 aattaaaaatgaacatttaaaaactct-----aggtaaactat-------   5401

human     3754 catacttagagttaactgttttgccaggctctgtatgcctactcataata   3803
                 |.|||||||||||.||||||||||.|.||||      ||.||||||||
mouse     5402 --tccttagagttaagtgttttgccaagttctg------taatcataata   5443

human     3804 tgataaaagcactcatctaatgctctgtaaatagaagtcagtgctttcca   3853
               |||||.||.|.||||...|....|||..|.||||||||.|.|.|||..||
mouse     5444 tgatagaaacgctcactcagcattctaaatatagaagttactccttcgca   5493

human     3854 tcagactgaactctcttgacaagatgtggatgaaattctttaagtaaaat   3903
               |.|.|||..|.| ||||||.|||.||
mouse     5494 tgacactctaat-tcttgataaggtg------------------------   5518

human     3904 tgtttactttgtcatacatttacagatcaaatgttagctcccaaagcaat   3953

mouse     5519 --------------------------------------------------   5518

human     3954 catatggcaaagataggtatatcatagtttgcctattagctgctttgtat   4003
                    |..|||||.||..|.|
mouse     5519 -----gagaaagagagagaga-----------------------------   5534

human     4004 tgctattattataaatagacttcacagttttagacttgcttaggtgaaat   4053
                                                    |....||.||.|.
mouse     5535 -------------------------------------gagggggagagac   5547

human     4054 tgcaattctttttactttcagtcttagataacaagtcttcaattatagta   4103
               .|.||.|                                  ||..|.||.
mouse     5548 agaaaat----------------------------------atggtggtt   5563

human     4104 caatcacacattgcttaggaatgcatcattaggcgatttgtcattatgc   4153
               |||..||...|||   |||.|      |||||
mouse     5564 caaggaccatttg---aggga------attag------------------   5586

human     4154 aaacatcatagagtgtacttacacaaacctagatagtatagcctttatgt   4203
                                                           ||||||
mouse     5587 --------------------------------------------ttatgt   5592

human     4204 acctaggccgtatggtatagtctgttgctcctaggccacaaacctgtaca   4253
               .|.|..|          |..|||||.|.||.||||
mouse     5593 tcttccg----------tcctctgtggatcttagg---------------   5617

human     4254 actgttactgtactgaatactatagacagttgtaacacagtggtaaatat   4303
                                           |.|..||.|||||
mouse     5618 ----------------------------ggttgaatacagt---------   5630

human     4304 ttatctaaatatatgcaaacagagaaaaggtacagtaaaagtatggtata   4353

mouse     5631 --------------------------------------------------   5630

human     4354 aaagataatggtatacctgtgtaggccacttaccacgaatggagcttgca   4403
                                                    .||.|||||.|..
```

```
mouse       5631 -------------------------------------cattgagctcggt   5643

human       4404 ggactagaagttgctctgggtgagtcagtgagtgagtggtgaattaatgt   4453
                 |||             |||.||
mouse       5644 gga-------------tggctg----------------------------   5652

human       4454 gaaggcctagaacactgtacaccactgtagactataaacacagtacgctg   4503

mouse       5653 --------------------------------------------------   5652

human       4504 aagctacaccaaatttatcttaacagtttttcttcaataaaaaattataa   4553

mouse       5653 --------------------------------------------------   5652

human       4554 ctttttaactttgtaaacttttaatttttaactttaaaatacttagc   4603
                                                ..||.||.|||....|..|
mouse       5653 -------------------------------tcctgttgaaaggtctgcc   5671

human       4604 ttgaaacacaaatacattgtatagctatacaaaaatatttttctttgta   4653
                 ..|.|...||||
mouse       5672 cagcagagcaaa--------------------------------------   5683

human       4654 tccttattctagaagctttttctatttctatttaaatttttttttt   4703
                                                    ||.|.||||||.|||
mouse       5684 -----------------------------------tagacttttttattt   5698

human       4704 acttgttagtcgttttgttaaaaactaaaacacacacactttcacctag   4753
                 ||.||....||..|||......||.||||....||
mouse       5699 acatggacatccgtttgtgactaatctaatgttca---------------   5733

human       4754 gcatagacaggattaggatcatcagtatcactcccttccacctcactgcc   4803

mouse       5734 --------------------------------------------------   5733

human       4804 ttccacctccacatcttgtcccactggaaggtttttaggggcaataacac   4853
                       ||||..|..|..||.|||.|                    |||.
mouse       5734 ------ctcccaaagtaatcacacag--------------------acag   5757

human       4854 acatgtagctgtcacctatgataacagtgctttctgttgaatacctcctg   4903
                 |.|.||||||                                   ||||.
mouse       5758 agaggtagct-----------------------------------tcctt   5772

human       4904 aaggacttgcctgaggctgttttacatttaacttaaaaaaaaaaaaagta   4953
                 .||                   |||..|||.||||.|
mouse       5773 cag-------------------tactcttaccttaca-------------   5790

human       4954 gaaggagtgcactctaaaataacaataaaaggcatagtatagtgaataca   5003
                                                           |||||.|
mouse       5791 ------------------------------------------tgaatcc-   5797

human       5004 taaaccagcaatgtagtagtttattatcaagtgttgtacactgtaataat   5053
                              ||..|.|||.||||   .|.||.|.||||.|||...|
mouse       5798 -------------taccattttgttat---tttttttccactttaaatct   5831

human       5054 t---gtatgtgctatactttaaataacttgcaaaatagtactaagacctt   5100
                 |   .||||||.|.|...|||.|.||.|||||.|..|.|....|.||..|
mouse       5832 ttgattatgtgtttttaattagaaaatttgcatacaaatttccatacagt   5881

human       5101 atgatggttacagtgtcactaaggcaatagcatattttcaggtccattgt   5150
                 |||
mouse       5882 atg-----------------------------------------------   5884

human       5151 aatctaatgggactaccatcatatatgcagtctaccattgactgaaacgt   5200
                                                 ||..||||||||
mouse       5885 --------------------------------tagaattgactg------   5896

human       5201 tacatggcacataactgtatttgcaagaatgatttgttttacattaatat   5250

mouse       5897 --------------------------------------------------   5896

human       5251 cacataggatgtacctttttagagtggtatgtttatgtggattaagatgt   5300
                                              |||||...|||.|.||||||..
mouse       5897 -----------------------------tgtttgaatgggtgaagatcc   5917

human       5301 acaagttgagcaaggggaccaagagccctgggttctgtcttggatgtgag   5350
                 [illegible]
```

```
                    |||.||                |...||...|.||||...|||.|.||||
mouse         5918 acatgt----------------gtaaccctagctctggactggctctgag   5951

human         5351 cgtttatgttcttctcctcatgtctgttttctcattaaattcaaaggctt   5400
                   |.|.|.||.|||||||          ||||
mouse         5952 cttgtttgctcttctc----------tttt--------------------   5971

human         5401 gaacgggccctatttagcccttctgttttctacgtgttctaaataactaa   5450
                                                    |||||||.|.|||||.|
mouse         5972 ---------------------------------gtgttctgagtaactga   5988

human         5451 agcttttaaattctagccatttagtgtagaactctctttgcagtgatgaa   5500
                   |.||.||..|||.||||...|||||.|   .|.|.|||..|        |
mouse         5989 aactctttcattttagcagcttagtat---gcgcccttcac--------a   6027

human         5501 atgctgtattggtttcttggctagcatattaaatattttatctttgtct   5550
                   .||||||..||..|.||...|||.|||......|.|..||||.||...||
mouse         6028 ttgctgtgctgcctgctgcactaacattactcctttgcttatgttcccct   6077

human         5551 tgatacttcaatgtcgttttaaacatcaggatcgggcttcagtattctca   5600
                   |..|..||||.||||.||||||.||..||.|..||.|.||||||   .|.
mouse         6078 tcctgattcagtgtcattttaagcagtagtactggacctcagta---cct   6124

human         5601 taaccagagagttcactgaggatacaggactg------------------   5632
                   ||.||  .|||.|||||||||..|||||.|||
mouse         6125 tagcc--ggagctcactgaggtgacagggctgaggctctgctgctgtctt   6172

human         5633 --------------tttgcccattttttgttatggct----ccagacttg   5664
                                 |||.....||||.||.|||..||    ||||..|||
mouse         6173 ttgagcttacctctttttaatgttttatggtatttctgctgccaggtttg   6222

human         5665 tggtatttccatgtcttttttttttttttttttttttgaccttttagcggc   5714
                   .||..||    |||.||.||||.||||||.|||||||
mouse         6223 ggggttt----tgttttgttttgttttttgttttttg-------------   6255

human         5715 tttaaagtatttctgttgttaggtgttgtattacttttctaagattactt   5764
                                           .||.|.|||.|||||||.||..||.|
mouse         6256 ------------------------ttttttttaattttctaggaacacct   6281

human         5765 aacaaagcaccacaaactgagtggctttaaacaacagcaatttattctct   5814
                   |..|||    ||||||||..|...|||.|||.|.||||...||.|||.||
mouse         6282 agaaaa----cacaaactaggaaacttaaaagagcagcgtcttgttccct   6327

human         5815 cacaattctagaagctagaagtccgaaatcaaagt----gttgacagggg   5860
                     ...||||||||..|..|||.|..|...||..||    ||||.||||..
mouse         6328 --gcgttctagaaagtccaagcctaatgccagtgtcatggttgtcaggaa   6375

human         5861 catgatcttcaagagagaagact----------ctttccttgcctcttcc   5900
                   |||||.|.||     .||||.||          |.||.||||.|||..|
mouse         6376 catgagcctc-----tgaaggcttcttgggaaacctttcttgtctcaac-   6419

human         5901 tggcttctggtggttaccagcaatcctgagtgttcctttcttgccttgta   5950
                     .|.||||||||..|.|||.|.|||   .||.|.||.|||  |..|..|
mouse         6420 --acctctggtggcaagcagtagtcc---atggtactctct--ctgtcca   6462

human         5951 gtttcaacaatccagtatctgccttttgtcttcacatggctgtctaccat   6000
                   ...|||.||..|||||..|||||.|||.||||......||.|.|.|.|.|
mouse         6463 cggtcagcatcccagtccctgccctttatctttgtgcagccgaccagctt   6512

human         6001 ttgtct-ctgtgtctccaaat------------ctctctccttataaaca   6037
                   |..|.| .|.|||||||...|            |.|.|||||..|||.||
mouse         6513 tgctttagtctgtctccttctcaggtctccttccccgctcctcttaagca   6562

human         6038 cagcagttattggattaggccccactctaatccagtatgaccccatttta   6087
                   |||||||.||||||||||..||||..||...|..|.|||.|||  .|||.
mouse         6563 cagcagtcattggattagagcccatccttccctcggatggccc--atttg   6610

human         6088 acatgattacacttatttctagataaggtcacattcacgtacaccaaggg   6137
                   ||.|.|||..||.|||||.||..|||||||.||||.||.||||
mouse         6611 acctaattttacgtatttgtaactaaggtcccatttacttaca-------   6653

human         6138 ttaggaattgaacatatctttttggggacacaattcaacccacaagtgt   6187

mouse         6654 --------------------------------------------------   6653

human         6188 cagtctctagctgagcctttcccttcctgtttttctcctttttagttgct   6237
```

```
human          [illegible]
                         |.|.|||.|.|||||||||||||..||   |||||.||..
mouse          6654 ----------cagggccctccccttcctgttttgttc---tttagctgaa   6690

human          6238 atgggttaggggccaaatctccagtcatactagaattgcacatggactgg   6287
                    ||||.||.|.|.||||||.||||.||||...|....||||||.|...||.
mouse          6691 atggtttggagaccaaatatccaatcattacaattgtgcacaagctatgt   6740

human          6288 atatttgggaatactgcgggtctattctatg--agctttagtatgtaaca   6335
                    ..||||||...||.|...||...|||||.||  ....||.||||||.|||
mouse          6741 tcatttggaggtaataaaggctcattctttgcttctattggtatgtgaca   6790

human          6336 tttaatatcagtgtaaagaagcccttttttaagttatttctttgaatttc   6385
                    ||
mouse          6791 tt------------------------------------------------   6792

human          6386 taaatgtatgccctgaatataagtaacaagttaccatgtcttgtaaaatg   6435

mouse          6793 --------------------------------------------------   6792

human          6436 atcatatcaacaaacatttaatgtgcacctactgtgctagttgaatgtct   6485

mouse          6793 --------------------------------------------------   6792

human          6486 ttatcctgataggagataacaggattccacatctttgacttaagaggaca   6535

mouse          6793 --------------------------------------------------   6792

human          6536 aaccaaatatgtctaaatcatttggggttttgatggatatctttaaattg   6585
                             |.|||||.|||.|||||| ||||||.|||||||||||||.|
mouse          6793 ---------tttctaagtcacttgggg-tttgatagatatctttaaatgg   6832

human          6586 ctgaacctaatcattg--gtttcatatgtcattgtttag-----------   6622
                    ||||||||.||||.||  .|||..||||||..|||||||
mouse          6833 ctgaacctgatcactgttcttttgtatgtccctgtttagctattgcaagc   6882

human          6623 --------------------------------------------------   6622

mouse          6883 gttcggataatgtgagacctggaatgcagtgagacctgggatgcagggat   6932

human          6623 --------------------------------------------------   6622

mouse          6933 gtcgactatctgccccccaccatctcctgctgttgccaagacagagattg   6982

human          6623 --------------------------------------------------   6622

mouse          6983 ctttaagtggtgaatcacccttgttggcggctacctttgcttactgggat   7032

human          6623 --------------------------------------------------   6622

mouse          7033 aatattcttggtcctagagtaaggcatatttgggctccaaagacagacca   7082

human          6623 --------------------------------------------------   6622

mouse          7083 agtgcttctcagtgatggagaaataacttttcttgccaaccacactctaa   7132

human          6623 --------------------------------------------------   6622

mouse          7133 atggagaaattcttcgaaatgcagagagtggggctatagatgtaaaattt   7182

human          6623 --------------------------------------------------   6622

mouse          7183 tttgtcttatctgaaaaaggggtaattattgtttcattaatcttcgacgg   7232

human          6623 --------------------------------------------------   6622

mouse          7233 aaactggaatggagatcggagcacttatggactatcaattatactgccgc   7282

human          6623 --------------------------------------------------   6622

mouse          7283 agacagagctgagcttctacctcccacttcacagagtgtgtgttgacagg   7332

human          6623 --------------------------------------------------   6622

mouse          7333 ctaacacacattattcgaaaaggaagaatatggatgcataaggtaagggg   7382
```

```
human           6623 --------------------------------------------------  6622

mouse           7383 cttttgagcttgatcatggtagcctggccaatgaaagtttttttctggta  7432

human           6623 --------------------------------------------------  6622

mouse           7433 cagttacacttaagttttggaaattatatgctgctaacaccagacagctg  7482

human           6623 --------------------------------------------------  6622

mouse           7483 ttatgttgtgtctcctgggcacagaaagccctgctctcatgcctggggtc  7532

human           6623 --------------------------------------------------  6622

mouse           7533 ttcacagtcctaatggaaagtaagatcttataaacattgtgtctgagttt  7582

human           6623 --------------------------------------------------  6622

mouse           7583 gttctggaagctgtgactctaccttcttgttttcctttccctgtgtgact  7632

human           6623 --------------------------------------------------  6622

mouse           7633 ttgtcctttgcttacaacagtgcaaaagtataaatattctcagattttga  7682

human           6623 --------------------------------------------------  6622

mouse           7683 taagctgtcagccacacagccttagtaactaagctgctgtcccacgctcc  7732

human           6623 --------------------------------------------------  6622

mouse           7733 cagttctgtataacgaggatggaccaattagattctaaggagttattcct  7782

human           6623 --------------------------------------------------  6622

mouse           7783 ttcaatttgcaaatttagctaaaggaaatattgttttctcctgatattta  7832

human           6623 --------------------------------------------------  6622

mouse           7833 cattgcttttcattttcagcatatctaaagaacaaacctaattctccttc  7882

human           6623 --------------------------------------------------  6622

mouse           7883 ctactttctagtttaatataatcctaaaaatccattaaaacatgactaat  7932

human           6623 --------------------------------------------------  6622

mouse           7933 tctataaggcctctaacctacaaagggaagtagcattttgaaaagaatag  7982

human           6623 --------------------------------------------------  6622

mouse           7983 ttttctctattatacctattcatgcagacttccttccttatttctgacat  8032

human           6623 --------------------------------------------------  6622

mouse           8033 acttaacaaaaatcatttagattcaaacagtttagctgcaggtgatatta  8082

human           6623 --------------------------------------------------  6622

mouse           8083 cagacaagtaatcccagtgctctatctagtctgaggcaaaaggatttgag  8132

human           6623 --------------------------------------------------  6622

mouse           8133 ctcagtgccagcctgttctatctacctggtgagttccagtcccataaata  8182

human           6623 --------------------------------------------------  6622

mouse           8183 aacaaactaaaacaaccgttcctctgttcctcagatgcgagtcgatcttg  8232

human           6623 --------------------------------------------------  6622

mouse           8233 tttgatttaaatagtgtgtaattattttcttttgaagctgcaggtgttat  8282

human           6623 --------------------------------------------------  6622

mouse           8283 gtgggctgttttagactaaattctctctttactgtggagtaaagggtgct  8332
```

```
human             6623 --------------------------------------------------   6622

mouse             8333 gtgattgtatttcatgttctctgcgagagcttgaacttgttgggctaatc   8382

human             6623 --------------------------------------------------   6622

mouse             8383 gcttgtctccatcctgtctccccacctgcgtaaaaagtattttcctgtga   8432

human             6623 --------------------------------------------------   6622

mouse             8433 gctgtacatgatagagcatatctacattgaaaaatgaacgagcatcaaaa   8482

human             6623 ---------------------------------   6622

mouse             8483 tggatttgttaaagtaaattttctttttcttag   8515


#---------------------------------------
#---------------------------------------
</pre></body></html>
```
