# Supplementary material for: A comparative bioinformatic analysis of C9orf72
Source: PeerJ. 2018 Feb 19;6:e4391. doi: 10.7717/peerj.4391 (PMC5822839; doi:10.7717/peerj.4391)
Supplement: Figure S7 — Sequence alignment of Intron 1 sequences using EBI’s EMBOSS Needle (Rice, Longden & Bleasby, 2000). [file peerj-06-4391-s009.pdf]

```
<html><head></head><body><pre style="word-wrap: break-word; white-space: pre-wrap;">########################################
# Program: matcher
# Rundate: Wed 24 Jan 2018 13:23:50
# Commandline: matcher
#    -auto
#    -stdout
#    -asequence emboss_matcher-I20180124-132346-0343-57893890-pg.asequence
#    -bsequence emboss_matcher-I20180124-132346-0343-57893890-pg.bsequence
#    -datafile EDNAFULL
#    -gapopen 25
#    -gapextend 4
#    -alternatives 1
#    -aformat3 pair
#    -snucleotide1
#    -snucleotide2
# Align_format: pair
# Report_file: stdout
########################################

#=======================================
#
# Aligned_sequences: 2
# 1: human
# 2: mouse
# Matrix: EDNAFULL
# Gap_penalty: 25
# Extend_penalty: 4
#
# Length: 640
# Identity:     415/640 (64.8%)
# Similarity:   415/640 (64.8%)
# Gaps:          67/640 (10.5%)
# Score: 944
#
#
#=======================================

human           3250 TATAATAGTTCAATTTTCACAACGTGGTAAAAG--TTTCCCTATAATTCA   3297
                     ||||||..||.||.||||.|||...|.|.|||.  |||.|||||||.||.
mouse           4944 TATAATGCTTTAAGTTTCCCAATCAGCTTAAAAGCTTTTCCTATAAATCT   4993

human           3298 ATCAGATTTTGCTCCAGGGTTCAGTTCTGTTTTAGGAAATACTTTTATTT   3347
                     .|.|||||.|||||..|||.|||.|.|||.||.|.|||...|||||.|||
mouse           4994 TTAAGATTATGCTCTGGGGCTCAATACTGCTTCAAGAAGGGCTTTTCTTT   5043

human           3348 TCAGTTTAATGATGAAATATTAGAGTTGTAATATTGCCTTTATGATTATC   3397
                     |                                  |..||||..|||||.
mouse           5044 T----------------------------------GTATTTAGAATTATT   5059

human           3398 CACCTTTTTAACCTAAAAGA----ATGAAAGAAAAATATGTTTGCAATAT   3443
                     |||||||||||.|.|||.||    |||.||.|.|||||||||||||||.||
mouse           5060 CACCTTTTTAAACAAAAGGAGAAAATGGAATAGAAATATGTTTGCAACAT   5109

human           3444 AATTTTATGGTTGTATGTTAACTTAATTCATTATGTTGGCCTCCAGTTTG   3493
                     |||||||||..|.|.||||.|.||......||.|||.|||||.|||||||
mouse           5110 AATTTTATGACTATGTGTTTATTTCGCGTGTTCTGTGGGCCTGCAGTTTG   5159

human           3494 CTGTTGTTAGTTATGACAGCAGTAGTGTCATTACCATTTCAATTCAGATT   3543
                     |||.|||||.|.|.||||.||||.|...||.|||..||||.|.|||||||
mouse           5160 CTGCTGTTAATGAGGACAACAGTGGCACCAATACAGTTTCCACTCAGATT   5209

human           3544 ACATTCCTATATTTGATCATTGTAAACTGACTGCTTACATTGTATTAAAA   3593
                     |||||| |.|.||...|...||.||.|||.|..||. ||.||.....|||
mouse           5210 ACATTC-TCTGTTCCCTTTCTGAAAGCTGCCCTCTC-CACTGGGCCCAAA   5257

human           3594 ACAGTGGATATTTTAAAGAAGCTGTACGGCTTATATCTAGTGCTGTCTCT   3643
                     |.|||...|||.|||||.|||||||||..||||.||. |.....||||||
mouse           5258 AGAGTCAGTATCTTAAACAAGCTGTACAACTTAGATA-ACCATGGTCTCT   5306


human           3644 TAAGACTATTAAATTGATACAACATATTTAAAAGTAAATATTACCTAAAT   3693
                     |.||||||.|.||||||.|.|   ||||.|||||||||||||.||||.||.|
mouse           5307 TCAGACTAGTTAATTGACATA---TATTAAAAAGTAAATAGTACCAAAGT   5353

[illegible]
```

```
human              3694 GAATTTTTGAAATTACAAATACACGTGTTAAAACTGTCGTTGTGTTCAAC   3743
                        ||||||.||||||||.||||..||.|.|.||||||.|.|.|      ||.
mouse              5354 GAATTTCTGAAATTAAAAATGAACATTTAAAAACTCTAGGT------AAA   5397

human              3744 CATTTCTGTACATACTTAGAGTTAACTGTTTTGCCAGGCTCTGTATGCCT   3793
                        |..|||        |||||||||||.||||||||||.|.|||||
mouse              5398 CTATTC--------CTTAGAGTTAAGTGTTTTGCCAAGTTCTGT------   5433

human              3794 ACTCATAATATGATAAAAGCACTCATCTAATGCTCTGTAAATAGAAGTCA   3843
                        |.|||||||||||||.||.|.||||...|....|||..|.||||||||.|
mouse              5434 AATCATAATATGATAGAAACGCTCACTCAGCATTCTAAATATAGAAGTTA   5483

human              3844 GTGCTTTCCATCAGACTGAACTCTCTTGACAAGATGTGGA   3883
                        .|.|||..|||.|.|||..|.| ||||||.|||.||..||
mouse              5484 CTCCTTCGCATGACACTCTAAT-TCTTGATAAGGTGGAGA   5522


#---------------------------------------
#---------------------------------------
</pre></body></html>
```
