# Supplementary material for: A comparative bioinformatic analysis of C9orf72
Source: PeerJ. 2018 Feb 19;6:e4391. doi: 10.7717/peerj.4391 (PMC5822839; doi:10.7717/peerj.4391)
Supplement: Figure S8 — Sequences, represented by black lines, are vertically aligned along their start positions. Each matrix family is represented by one unique colour wherein matches to matrices of the same family are painted the same colour. Matches found on the positive or negative strand are shown on top or below the sequence line, respectively. Transcription factors were identified by the program MatInspector (Cartharius et al., 2005). [file peerj-06-4391-s010.pdf]

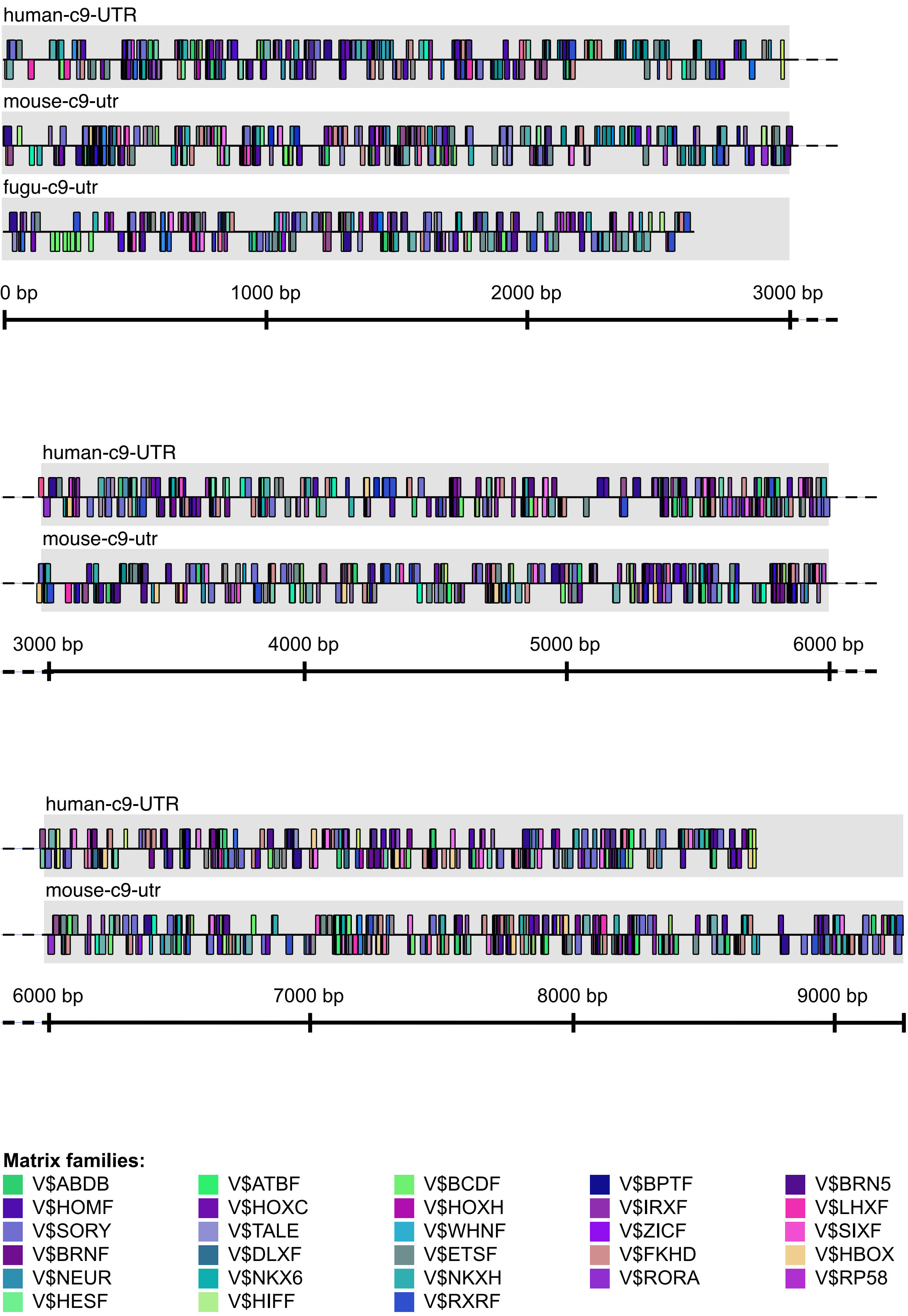

human-c9-UTR
mouse-c9-utr
fugu-c9-utr
0 bp
1000 bp
2000 bp
3000 bp
human-c9-UTR
mouse-c9-utr
3000 bp
4000 bp
5000 bp
6000 bp
human-c9-UTR
mouse-c9-utr
6000 bp
7000 bp
8000 bp
9000 bp
Matrix families:
V$ABDB
V$ATBF
V$BCDF
V$BPTF
V$BRN5
V$HOMF
V$HOXC
V$HOXH
V$IRXF
V$LHXF
V$SORY
V$TALE
V$WHNF
V$ZICF
V$SIXF
V$BRNF
V$DLXF
V$ETSF
V$FKHD
V$HBOX
V$NEUR
V$NKX6
V$NKXH
V$RORA
V$RP58
V$HESF
V$HIFF
V$RXRF
