# Supplementary material for: A comparative bioinformatic analysis of C9orf72
Source: PeerJ. 2018 Feb 19;6:e4391. doi: 10.7717/peerj.4391 (PMC5822839; doi:10.7717/peerj.4391)
Supplement: Figure S5 — Sequence alignment of Intron 1 sequences using EBI’s EMBOSS Matcher (Rice, Longden & Bleasby, 2000). [file peerj-06-4391-s007.pdf]

```
<html><head></head><body><pre style="word-wrap: break-word; white-space: pre-wrap;">########################################
# Program: matcher
# Rundate: Wed 24 Jan 2018 13:29:12
# Commandline: matcher
#    -auto
#    -stdout
#    -asequence emboss_matcher-I20180124-132911-0542-39055053-p1m.asequence
#    -bsequence emboss_matcher-I20180124-132911-0542-39055053-p1m.bsequence
#    -datafile EDNAFULL
#    -gapopen 10
#    -gapextend 4
#    -alternatives 1
#    -aformat3 pair
#    -snucleotide1
#    -snucleotide2
# Align_format: pair
# Report_file: stdout
########################################

#=======================================
#
# Aligned_sequences: 2
# 1: human
# 2: fugu
# Matrix: EDNAFULL
# Gap_penalty: 10
# Extend_penalty: 4
#
# Length: 603
# Identity:     327/603 (54.2%)
# Similarity:   327/603 (54.2%)
# Gaps:         102/603 (16.9%)
# Score: 189
# 
#
#=======================================

human            798 TGCCGGCGAGGAGATC-ATGCGGGATGAGATGGGGGTGTGGAGACGCC--    844
                     |.|.||||.|||.||. |.|..|.|||..|..|.|||.|| ||....|
fugu              34 TTCAGGCGGGGACATAGAAGTCGCATGCAAACGTGGTTTG-AGCTTTCAT     82

human            845 TGCACAATTTCAGCCCAAGC---TTCTAGAGAGTGGTGATGACTTGCATA    891
                     |.|.|. ||..||.|..|.|   ||.|..|..|.|| |||   |.|.|.|
fugu              83 TTCTCC-TTATAGACTGACCCCTTTTTTCAATGCGG-GAT---TGGAAGA    127

human            892 -TGAGGGCAGCAATGCAAGTCG-GTGTGCTCCCCATTCTGTGG-GAC-AT    937
                      |.|||.||..|||  ||.||| |||.|.||.||  ||.|||. ||. |.
fugu             128 GTAAGGACAATAAT--AACTCGTGTGAGGTCTCC--TCAGTGAAGAGGAG    173

human            938 GACCTGGTTGCTTCACAGCTCCGAGATGA---CACAGAC--TTGCT-TAA    981
                     |.|.||  ||.| ||.|||...|||||||   |...|.|  ||||| ||.
fugu             174 GGCATG--TGAT-CATAGCCAAGAGATGAATGCGTTGGCCTTTGCTGTAT    220

human            982 -AGGAAGTGACTATTG-TGACTTGG-G-----CATCACTTGACTGATGGT   1023
                      ||||||||..|...| |||..||| |     |||||.|.|..|||||..
fugu             221 TAGGAAGTGGTTGCAGATGAGATGGTGGTTGTCATCAGTGGTTTGATGAC    270

human           1024 A--ATCAGT--TGTCTAAAGAAGTGCACAG-ATTACATGTCCGTG-TGCT   1067
                     |  ||||..  ||.|   |....|..|||. |||.||||.|..|| || |
fugu             271 ATCATCAAACCTGCC---ATTTTTCAACATCATTTCATGGCTTTGGTG-T    316

human           1068 CATTGGGTCTAT-CTGGCCGCGTTGAACACCACCAGGCTTTGTATTCAGA   1116
                     |.|.|...||.| |.||||.|.|....|..|.||.|.|||||. |||.||
fugu             317 CCTGGACACTGTTCAGGCCACTTCCTTCTTCCCCTGACTTTGC-TTCTGA    365

human           1117 AACAGGAGGGAGGTCCTGCACTTTC-CCAGGAGGGGTGGCCCTTTCAGAT   1165
                     ||..|.|.. ||...|...|.|.|. |||..     ||   |..|.|.|.
fugu             366 AAGTGTAAA-AGCAACAAAAATATGTCCATC-----TG---CAATGAAAA    406


human           1166 GCAATCGA-GATTGTTAGGCTCTGGGAGAGTAGTTGCCTGGTTGTGGCAG   1214
                     ||||..|| ||.. ||.|..||||  ||| |.|  |||..|.    |||.
fugu             407 GCAAGTGATGAGC-TTTGTTTCTG--AGA-TCG--GCCAAGA----GCAC    446

[illegible]
```

```
human           1215 TTGGTAAATTTCTATTCAAACAGTTGCCATGCACCAGTTGTTCACAACAA   1264
                     | |||   ||||    |.|| |||| |.|||        |..| ||||||
fugu             447 T-GGT---TTTC----CTAA-AGTT-CTATG--------GGAC-CAACAA    477

human           1265 GGGTA-CGTAATCTGTCTGGCATTACTTCTA-CTT-T-TGTACAAAGGAT   1310
                     |  || ||.|||||..||. | ||..||.|| ||| | |||.|.||.|||
fugu             478 G--TAGCGGAATCTTGCTT-C-TTCTTTATAACTTATATGTCCTAAAGAT    523

human           1311 ---CAAAAAAAAAAAAGATACTG-TT-AAGATATGATTTTTCTCAGACTT   1355
                        ||.|........|||.|||| || ||...||..||||.||||.|.||
fugu             524 ACTCATATCCTTGTCAGAAACTGCTTGAATCAATTGTTTTCCTCACATTT    573

human           1356 TGG   1358
                     |.|
fugu             574 TAG    576


#---------------------------------------
#---------------------------------------
</pre></body></html>
```
